# The proportion of missing data should not be used to guide decisions on multiple imputation

**DOI:** 10.1016/j.jclinepi.2019.02.016

**Published:** 2019-06

**Authors:** Paul Madley-Dowd, Rachael Hughes, Kate Tilling, Jon Heron

**Affiliations:** a Population Health Sciences, Bristol Medical School, University of Bristol, Oakfield House, Oakfield Grove, Bristol BS8 2BN, UK; b MRC Integrative Epidemiology Unit, University of Bristol, Oakfield House, Oakfield Grove, Bristol BS8 2BN, UK

**Keywords:** ALSPAC, Bias, Methods, Missing data, Multiple imputation, Simulation

## Abstract

**Objectives:**

Researchers are concerned whether multiple imputation (MI) or complete case analysis should be used when a large proportion of data are missing. We aimed to provide guidance for drawing conclusions from data with a large proportion of missingness.

**Study Design and Setting:**

Via simulations, we investigated how the proportion of missing data, the fraction of missing information (FMI), and availability of auxiliary variables affected MI performance. Outcome data were missing completely at random or missing at random (MAR).

**Results:**

Provided sufficient auxiliary information was available; MI was beneficial in terms of bias and never detrimental in terms of efficiency. Models with similar FMI values, but differing proportions of missing data, also had similar precision for effect estimates. In the absence of bias, the FMI was a better guide to the efficiency gains using MI than the proportion of missing data.

**Conclusion:**

We provide evidence that for MAR data, valid MI reduces bias even when the proportion of missingness is large. We advise researchers to use FMI to guide choice of auxiliary variables for efficiency gain in imputation analyses, and that sensitivity analyses including different imputation models may be needed if the number of complete cases is small.

**What is new?**

**Key findings**
• Unbiased results can be obtained even with large proportions of missing data (up to 90% shown in our simulation study), provided the imputation model is properly specified and data are missing at random.
• The fraction of missing information was better as a guide to the efficiency gains from MI than the proportion of missing data.

**What this adds to what was known?**
• The proportion of missing data provides limited information about the bias and efficiency gains that can be made from multiple imputation.
• Increasing the number of auxiliary variables included in an imputation model does not always result in efficiency gains.

**What is the implication and what should change now?**
• The proportion of missing data should not be used as a guide to inform decisions about whether to perform multiple imputation or not. The fraction of missing information should be used to guide the choice of auxiliary variables in imputation analyses.

## 1 Introduction

Missing data is a common problem in epidemiology, and participant drop out can substantially reduce the sample size available for analysis even in initially large cohorts. Missing data (also referred to as missingness) may cause bias and will always cause a reduction in efficiency. Analyses that account for missing data must consider the reasons for missingness (known as a missingness mechanism). Using Rubin's terminology [1], reasons for missing data are classified as missing completely at random (MCAR) where the probability of missingness does not depend on either observed or missing data, missing at random (MAR) where conditional on the observed data, the probability of missingness is independent of unobserved data, and missing not at random (MNAR), where the probability of missingness is dependent on unobserved data even after conditioning on observed data. Readers may wish to refer to the studies by Graham [2] and Donders et al [3] for intuitive explanations of these terms.

A common approach [4] (and the default in most statistical packages) for dealing with missing data is complete case analysis (CCA), which restricts the analysis to individuals with complete data. An alternative to CCA is multiple imputation (MI) [5], [6], which creates m copies of the data set, replacing the missing values in each data set with independent random draws from the predictive distribution of the missing values under a specific model (the imputation model). The analysis model is then fitted to each imputed data set and the multiple results are combined into one inference using Rubin's rules [5]. The imputation model should contain all variables in the analysis model [7], [8], [9] and any interactions between variables [10]. The imputation model can additionally include variables not included in the analysis model, which are known as auxiliary variables. These are included to make the MAR assumption (required in the standard implementation of MI to produce unbiased estimates) more plausible and to provide information about the missing values [11].

Researchers in a variety of fields often ask what proportion of missing data warrants the use of MI [12], [13], [14], [15]. Varying guidance exists; in the literature, 5% missingness has been suggested as a lower threshold below which MI provides negligible benefit [16]. In contrast, one online tutorial has stated that 5% missing data is the maximum upper threshold for large data sets [17]. Statistical guidance articles have stated that bias is likely in analyses with more than 10% missingness and that if more than 40% data are missing in important variables then results should only be considered as hypothesis generating [18], [19].

The above suggested cutoff points, with respect to specified proportions of missing data, have a limited evidence base to support them. A small number of studies have investigated bias and efficiency in data sets with increasing proportions of missing data. This has commonly been done with a maximum of 50% missing data in studies that showed increasing variability of effect estimates with increased missingness [20], [21], [22]; mixed results were found for bias. Where more than 50% missingness has been investigated, the use of auxiliary variables has often not been examined [23], [24]. Evidence of how varying quantities of missing data and auxiliary information jointly affect estimates obtained from MI is lacking in the literature as a result. The influence of the proportion of missing data on bias and efficiency (measured jointly using mean squared error) was shown to depend on the type of missingness (MCAR, MAR or MNAR) [23] and which variable (outcome, exposure, or confounder) is missing [24]. Where both more than 50% missingness and auxiliary variables have been used, the study sample size was very small (*N* ≤ 200), thus limiting the applicability of results to larger epidemiological studies [25].

The proportion of missing data is a common measure of how much information has been lost because of missing values in a data set. However, it does not reflect the information retained by auxiliary variables. Alternative measures such as the fraction of missing information (FMI) may be more useful as a tool for determining potential efficiency gains from MI. The FMI is a parameter-specific measure that is able to quantify the loss of information due to missingness, while accounting for the amount of information retained by other variables within a data set [11], [26]. The FMI, derived from MI theory [5], [27], can be interpreted as the fraction of the total variance (including both between and within imputation variance, see Supplementary material) of a parameter, such as a regression coefficient, that is attributable to between imputation variance, for large numbers of imputations m. Values of FMI range between 0 and 1. A large FMI (close to 1) indicates high variability between imputed data sets; that is, the observed data in the imputation model do not provide much information about the missing values.

In this article, we have conducted a simulation study to show (1) that MI can be used to provide unbiased estimates with improved efficiency compared to CCA at any proportion of missing data and (2) the utility of the FMI as a guide to the likely efficiency gains from using MI. We then use an applied example to show the influence of auxiliary information on the FMI, examining the association between maternal smoking during pregnancy and offspring intelligence quotient (IQ) score at age 15 using the Avon Longitudinal Study of Parents and Children (ALSPAC). Finally, we present a discussion of our findings and our conclusions.

## 2 Simulation study

### 2.1 Methods

Via simulations, we compare FMI and the proportion of missing data to measure gain in information from MI compared with CCA, in scenarios with different available auxiliary information and amounts of missing data. Our simulated data sets are motivated by a prospective cohort study where all baseline data are available but some follow-up data are missing.

#### 2.1.1 Data model

We simulated data from a multivariate normal distribution where all variables had a mean of 0 and a standard deviation of 1. Each simulated data set contained 1,000 observations on continuous variables outcome Y, exposure X, and auxiliary variables $Z_{1}-Z_{11}$. All variables were correlated with Y and all variables except Y had zero correlation with each other. The correlation between Y and X was 0.6, Y and $Z_{1}-Z_{2}$ was 0.4, Y and $Z_{3}-Z_{7}$ was 0.2, and finally between Y and $Z_{8}-Z_{11}$ was 0.1.

Missingness was simulated under an MCAR mechanism to examine the benefit of MI to improve efficiency in the absence of bias and an MAR mechanism to further examine bias reduction. The MCAR missingness mechanism removed the first p observations such that $\frac{p}{n}$ gives the required proportion of missing data. MAR missingness was simulated under a logistic regression model using

$$\text{logit}\left({\text{λ}}_{i}\right)=\text{α}+{\text{Z}}_{1i}+X_{i}$$

The value of α was manipulated for the different simulation settings to provide the required proportion of missing data on average across data sets.

#### 2.1.2 Analysis model

For each simulation setting and imputation model, the following linear regression analysis model was used:

$$y_{i}=\beta_{0}+\beta_{1}x_{i}+\;\varepsilon_{i},$$

where $\beta_{0}$ (true value equal to 0) and $\beta_{1}$ (true value equal to 0.6) are the intercept and exposure coefficient, respectively, and $\varepsilon_{i}$ are independently and identically distributed random errors with distribution $N\left(0,\;\sigma^{2}\right)$.

Each simulated data set was analyzed using CCA and MI. Where data were simulated as MCAR, both MI and CCA are valid models [28]. For MAR data, with missingness dependent on X and ${\text{Z}}_{1}$, CCA is biased unless both X and ${\text{Z}}_{1}$ are included in the analysis model. For MAR data, MI is valid provided both X and ${\text{Z}}_{1}$ are included in the imputation model. MI was performed using the Stata [29] package *mi impute*. The analysis model, and the combination across imputed data sets using Rubin's rules, was implemented via Stata's *mi estimate.*

#### 2.1.3 Imputation models

Five imputation models were considered for both MCAR and MAR data (see Table 1). All models contained the variables included in the analysis model and used linear regression to impute the missing outcome. Model 1 contained no auxiliary information. Models 2–5 contained increasing quantities of auxiliary information, achieved by increasing the number of Z variables included in the imputation model. The squared coefficient of multiple correlation with the outcome variable, ${R_{Y}}^{2}$, was used as a measure of the quantity of auxiliary information. This reflects a sum of the independent contributions of each auxiliary variable to the imputation model.

**Table 1:** Description of the imputation models used for both MCAR and MAR data

Imputation model	Variables included	${R_{Y}}^{2}$^a^
1 (least auxiliary information)	*Y, X*	0.36
2	*Y, X, Z*_*3*_	0.40
3	*Y, X, Z*_*1*_	0.52
4	*Y, X, Z*_*1–4*_	0.76
5 (most auxiliary information)	*Y, X, Z*_*1–11*_	0.92

a ${R_{Y}}^{2}$, the total coefficient of multiple correlation with the outcome Y for all variables included in the imputation model, is displayed as a measure of the strength of the auxiliary information in each imputation model.

For each imputation model, 1,000 imputations were run. FMI is a highly variable estimate at low numbers of imputations [30], hence the need for a large number of imputations. See Figure S1 in the supplementary material on why we chose 1,000 imputations.

#### 2.1.4 Comparisons

We repeated the simulation study for 1%, 5%, 10%, 20%, 40%, 60%, 80%, and 90% missing data. For all scenarios, we generated 1,000 independent simulated data sets. Separately for the exposure coefficient and the constant coefficient, we compared the CCA and MI analyses with respect to the bias, empirical standard error (SE), and FMI of the coefficient estimates. Bias and empirical SE were estimated using the *simsum* command in Stata [31], and FMI was calculated using Stata's *mi estimate*. We report the median value and interquartile range of the FMI across simulations. Further measures are described and presented in the Supplementary material along with formulae for all performance statistics.

### 2.2 Results

**Fig. 1:**
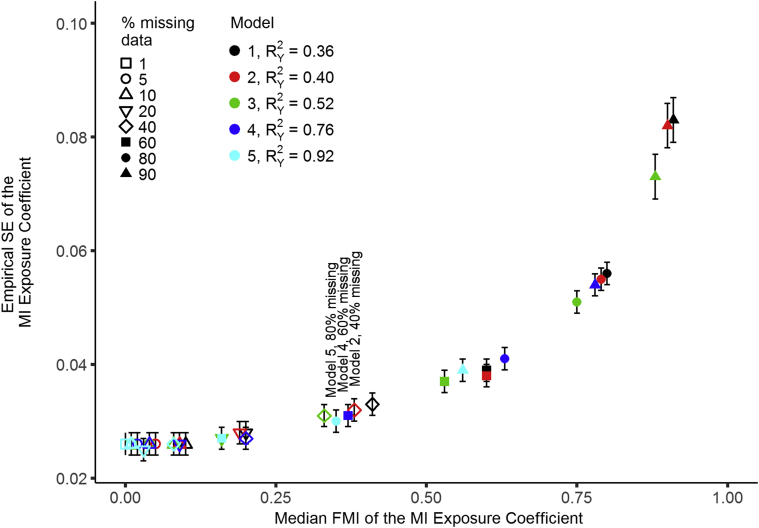
Empirical SE of the MI exposure coefficient plotted against FMI for simulated MCAR data. Error bars are 95% confidence intervals based on Monte Carlo standard errors across simulations. FMI = fraction of missing information; MCAR = missing completely at random; MI = multiple imputation; SE = standard error.

Figure 1 displays the empirical SE of the MI exposure coefficient against the FMI, according to proportions of missing data (see Supplementary Figure S2 for presentation of the data separated by panels of percentage missing data), which demonstrates that for any given proportion of missing data, the empirical SE increases as the FMI increases–with this association being most noticeable at high proportions of missing data. For every value of the proportion of missing data, the FMI for models with no auxiliary information was approximately equal to the proportion of missing data. The FMI decreased with increasing quantities of auxiliary information. For different proportions of missing data but similar FMI values, the empirical SE of MI coefficient estimates was approximately the same. For example, compare model 2 for 40% missing data (FMI = 0.38, empirical SE = 0.032) with model 4 for 60% missing data (FMI = 0.37, empirical SE = 0.031) and model 5 for 80% missing data (FMI = 0.35, empirical SE = 0.030). A second example is given by the comparison of model 1 for 60% missing data (FMI = 0.60, empirical SE = 0.039), model 4 of 80% missing data (FMI = 0.63, empirical SE = 0.041), and model 5 of 90% missing data (FMI = 0.56, empirical SE = 0.039), and a third example is given by model 2 for 80% missing data (FMI = 0.79, empirical SE = 0.055) and model 4 for 90% missing data (FMI = 0.78, empirical SE = 0.054). This indicates that the FMI is a good measure of estimate precision, whereas the proportion of missing data is not.

Table 2 displays the percentage reduction in empirical SE compared to CCA for each MI model. Increasing auxiliary information in the imputation model led to increasing gains in efficiency (greater reduction in empirical SE) with greater effects seen at larger proportions of missing data. For low proportions of missing data, there was little efficiency gain from MI even for the model with the largest quantity of added auxiliary information.

**Table 2:** Percentage reduction in empirical SE and bias compared with CCA for MCAR and MAR results of the exposure coefficient in the simulation study

% Missing	Imputation model^a^^,^^b^	% Reduction in SE compared to CCA^c^		% Reduction in bias compared to CCA^d^
		MCAR data	MAR data	MAR data
1	1: R^2^ = 0.36 (No aux info)	0.00%	−0.01%	1.46%
	2: R^2^ = 0.40	0.16%	0.24%	1.91%
	3: R^2^ = 0.52	0.24%	0.11%	79.03%
	4: R^2^ = 0.76	0.55%	0.41%	79.54%
	5: R^2^ = 0.92	0.52%	0.58%	81.42%
5	1: R^2^ = 0.36 (No aux info)	0.02%	−0.03%	0.16%
	2: R^2^ = 0.40	0.19%	0.03%	−1.26%
	3: R^2^ = 0.52	1.04%	0.93%	97.92%
	4: R^2^ = 0.76	1.99%	2.63%	94.91%
	5: R^2^ = 0.92	1.57%	3.64%	93.74%
10	1: R^2^ = 0.36 (No aux info)	−0.05%	−0.06%	0.40%
	2: R^2^ = 0.40	0.37%	0.75%	−0.35%
	3: R^2^ = 0.52	0.58%	1.12%	97.38%
	4: R^2^ = 0.76	2.59%	4.61%	96.73%
	5: R^2^ = 0.92	2.89%	6.76%	96.41%
20	1: R^2^ = 0.36 (No aux info)	0.03%	−0.05%	−0.19%
	2: R^2^ = 0.40	1.08%	1.03%	−0.65%
	3: R^2^ = 0.52	2.59%	3.42%	97.94%
	4: R^2^ = 0.76	8.28%	7.94%	97.33%
	5: R^2^ = 0.92	10.53%	10.26%	97.29%
40	1: R^2^ = 0.36 (No aux info)	0.05%	−0.06%	−0.21%
	2: R^2^ = 0.40	2.00%	1.25%	0.10%
	3: R^2^ = 0.52	5.37%	5.06%	97.84%
	4: R^2^ = 0.76	15.56%	14.11%	98.56%
	5: R^2^ = 0.92	21.10%	22.86%	98.64%
60	1: R^2^ = 0.36 (No aux info)	−0.04%	−0.02%	0.21%
	2: R^2^ = 0.40	2.55%	1.68%	0.02%
	3: R^2^ = 0.52	5.48%	6.74%	99.77%
	4: R^2^ = 0.76	21.02%	18.45%	99.43%
	5: R^2^ = 0.92	31.59%	31.96%	98.22%
80	1: R^2^ = 0.36 (No aux info)	−0.03%	−0.14%	0.00%
	2: R^2^ = 0.40	2.16%	1.57%	1.34%
	3: R^2^ = 0.52	8.18%	9.86%	96.47%
	4: R^2^ = 0.76	27.56%	28.21%	99.62%
	5: R^2^ = 0.92	45.88%	44.66%	98.77%
90	1: R^2^ = 0.36 (No aux info)	0.03%	0.11%	0.04%
	2: R^2^ = 0.40	1.40%	2.18%	0.89%
	3: R^2^ = 0.52	12.44%	8.86%	99.97%
	4: R^2^ = 0.76	34.82%	33.76%	95.78%
	5: R^2^ = 0.92	53.09%	52.96%	98.73%

*Abbreviations*: CCA, complete case analysis; MAR, Missing at random; MCAR, Missing completely at random; SE, Standard error.

a R^2^ refers to the squared coefficient of multiple correlation which is used as a measure of auxiliary information.

b Models 1 and 2 do not include all variables in the missingness mechanism and so are biased (as expected) for the MAR data. Models 3–5 do include all variables in the missingness mechanism and so are unbiased (as expected).

c Calculated using 100 × (se_CCA_–se_MI_)/se_CCA_, where se_CCA_ and se_MI_ are the empirical standard error of the CCA model and the MI model, respectively.

d Calculated using 100 × (abs(bias_CCA_)-abs(bias_MI_))/abs(bias_CCA_), where abs(.) is a function giving the absolute value and bias_CCA_ and bias_MI_ are the bias of the CCA model and the MI model, respectively.

**Fig. 2:**
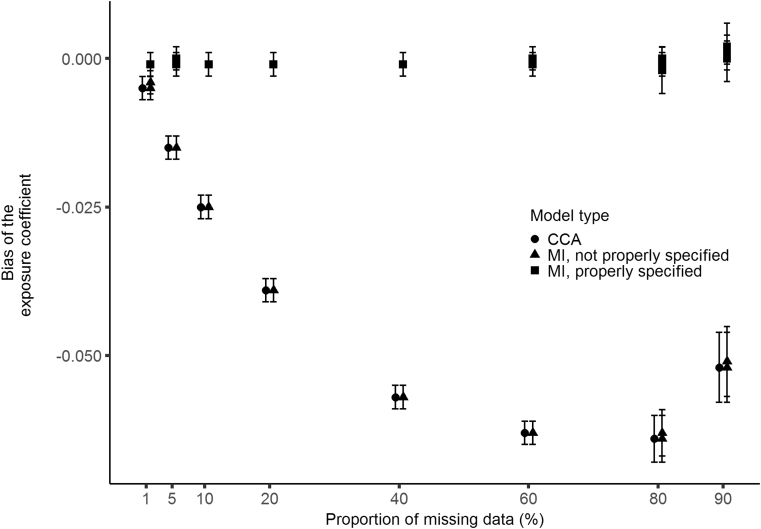
Bias of the CCA and MI exposure coefficient plotted against the proportion of missing data for simulated MAR data. Error bars are 95% confidence intervals based on Monte Carlo standard errors across simulations. CCA = complete case analysis; MI = multiple imputation; FMI = fraction of missing information; SE = standard error.

Figure 2 shows that for CCA there are increasing levels of bias in estimating the exposure coefficient with increasing proportions of missing data. A single exception to this occurs at 90% missing data, which may be due to increased variability of the estimate. For MI, no bias was observed at any proportion of missing data, provided the imputation model included all variables related to missingness (models 3–5). These findings provide an example of valid estimates from properly specified MI at much larger proportions of missing data than current guidance [19] advises. When the imputation model did not include these variables (models 1-2) then the magnitude of bias was similar to that of CCA. Data for the constant coefficient are presented as supplementary material in Table S1.

All performance statistics for the exposure coefficient across simulations of MCAR and MAR data are presented in Supplementary Table S2 and S3, respectively. The results for the constant coefficients of the MCAR and MAR data are presented in Table S4 and S5. With respect to FMI and efficiency of the MI estimates, the results for the MAR scenario followed the same patterns as noted for the MCAR scenario. The results of FMI and efficiency gains were similar when missingness depended on the auxiliary variable and when missingness did not depend on the auxiliary variable (see Supplementary Table S6).

## 3 Applied example

### 3.1 Ethical approval

Ethical approval for the study was obtained from the ALSPAC Ethics and Law Committee and the Local Research Ethics Committees - http://www.bristol.ac.uk/alspac/researchers/research-ethics/.

### 3.2 Methods

Data were taken from ALSPAC [32], [33] which recruited 14,541 pregnant women residents in Avon, UK, with expected dates of delivery from 1st April 1991 to 31st December 1992. Of these pregnancies, there were 13,988 children who were alive at 1 year of age. Please note the study website contains details of all the data that are available through a fully searchable data dictionary (http://www.bristol.ac.uk/alspac/researchers/our-data/).

We investigated the relationship between a binary measure of maternal smoking during pregnancy, self-reported at 18 weeks gestation and offspring IQ measured using the Wechsler Abbreviated Scale of Intelligence at age 15 years [34]. The substantive analysis was a linear regression of offspring IQ at age 15 years on maternal smoking in pregnancy. We shall refer to this as the “unadjusted” analysis. We also considered an “adjusted” analysis which controlled for the possible confounders maternal age, parity and education, and offspring sex.

To simplify this illustrative example, observations were removed if they had missing data for any of the confounders. Our justification for this decision is that these variables were measured at the start of the study and if they were missing then the participant was likely to be missing data in most other variables. Table S7 shows excluded participants with missing values in the confounders were more likely to have a larger number of missing variables for the outcome, exposure, and auxiliary variables. This exclusion criteria left a total sample size of n=11911. Among the included participants, the exposure was fully observed. See Table S8 for the patterns of missing data for the outcome and auxiliary variables.

The auxiliary variables used in imputation models were IQ at age of 8 years measured using the Wechsler Intelligence Scale for Children–III [35], intelligibility and fluency at age of 9 years measured using the Children's Communication Checklist [36], a binary indicator of ever having learning difficulties, and, measured in school year 6, the child's teacher-reported maths and literacy streaming groups as well as the score from a maths assessment.

We performed chained equations imputation [37] using Stata's *mi impute chained* command with 1,000 imputations. We used this large number of imputations to ensure that a reliable estimate of the FMI was obtained. Twelve imputation models with differing amounts of auxiliary information were investigated. A description of the variables included in each model is displayed in Table 3. Model A contains only the confounders in the adjusted model and models B–E include one auxiliary variable each. Model F includes one variable each for the maths and literacy streaming groups. Models G–L include differing combinations of auxiliary variables.

**Table 3:** Imputation models for the applied example, Bristol, United Kingdom, 1991–2007

Model	Variables included^a^	% Missing data
A	No extra variables	62.47%
B	IQ at age 8	66.64%
C	Intelligibility and fluency at age 9	66.68%
D	Maths assessment score	76.59%
E	Learning difficulties	78.84%
F	Streaming for maths and English	81.75%
G	IQ at age eight and intelligibility	69.34%
H	IQ at age eight and maths assessment	79.11%
I	IQ at age 8, intelligibility, and maths assessment	80.62%
J	IQ at age 8, intelligibility, maths assessment and LD	84.17%
K	IQ at age 8, intelligibility, maths assessment and streaming groups	86.42%
L	IQ at age 8, intelligibility, maths assessment, LD, and streaming groups	86.51%

*Abbreviations*: IQ, intelligence quotient; LD, learning difficulties.

a All models additionally contained IQ at the age of 15 years, a binary measure of maternal smoking in pregnancy and the set of all confounders. Continuous variables (IQ at age of 8 and 15 years, intelligibility, and maths assessment score) were imputed using a linear regression model, binary variables (sex and learning difficulties) were imputed using logistic regression, and ordinal variables (maternal age and education, parity, and maths and literacy streaming group) were imputed using ordinal logistic regression.

The same imputation models were used for the unadjusted and adjusted analyses. For a given analysis model, an imputation model was defined as containing auxiliary variables if it included variables that were not in the analysis model. So, for the unadjusted analysis, every imputation model contained auxiliary variables, whereas for the adjusted analysis, the simplest imputation model contained no auxiliary variables.

### 3.3 Results

Table 4 shows that the proportion of missing data in the outcome variable was 62%, with all auxiliary variables having a lower proportion of missing data. IQ at age of eight years and maths assessment score explained the most variance in the outcome. Intelligibility and ever having a learning disability were the weakest predictors. The exposure and all confounder and auxiliary variables were associated with the likelihood of missingness in the outcome variable.

**Table 4:** Variable description, including the proportion of missing data and relationship with observed and missing values in the outcome variable for the applied example, Bristol, United Kingdom, 1991–2007

Variable	Type	% Missing data	$R^{2}$ with Outcome^a^	OR for missing data in outcome^b^	95% CI^b^
IQ at age 15	Continuous	62.47			
Maternal smoking in pregnancy	Binary	0.00	0.01	2.18	1.98, 2.39
Maternal age	Categorical	0.00	0.04		
	≤ 24 years			Reference	Reference
	25–29 years			0.57	0.51, 0.64
	30–34 years			0.42	0.38, 0.47
	≥ 35 years			0.41	0.35, 0.47
Parity	Categorical	0.00	0.01		
	0			Reference	Reference
	1			1.18	1.09, 1.29
	2			1.46	1.30, 1.64
	≥ 3			2.06	1.72, 2.48
Sex	Binary	0.00	<0.01		
	Female			Reference	Reference
	Male			1.27	1.18, 1.37
Maternal education	Categorical	0.00	0.11		
	Vocational			Reference	Reference
	CSE/O level			0.91	0.80, 1.05
	A level/degree			0.45	0.39, 0.52
IQ at age 8	Continuous	44.49	0.37	0.98	0.98, 0.98
Intelligibility and fluency at age 9	Continuous	37.96	0.01	0.95	0.93, 0.97
Maths assessment score	Continuous	44.39	0.24	0.15	0.12, 0.19
Ever had learning difficulties	Binary	48.57	0.08	2.02	1.75, 2.33
Maths streaming group	Ordinal	52.76	0.20		
	Lowest			Reference	Reference
	Middle			0.58	0.50, 0.69
	Highest			0.42	0.36, 0.49
Literacy streaming group	Ordinal	55.03	0.16		
	Lowest			Reference	Reference
	Middle			0.59	0.50, 0.69
	Highest			0.39	0.33, 0.45

*Abbreviations*: CCA, complete case analysis; CI, confidence interval; IQ, Intelligence quotient; OR, odds ratio; R^2^, variance explained in the outcome.

a Regressed IQ at the age of 15 years, on each variable with no adjustment for other variables. CCA was used in all models.

b Using logistic regression, the odds of having a missing value for the outcome were regressed on each variable with no adjustment for other variables. CCA was used in all models.

**Fig. 3:**
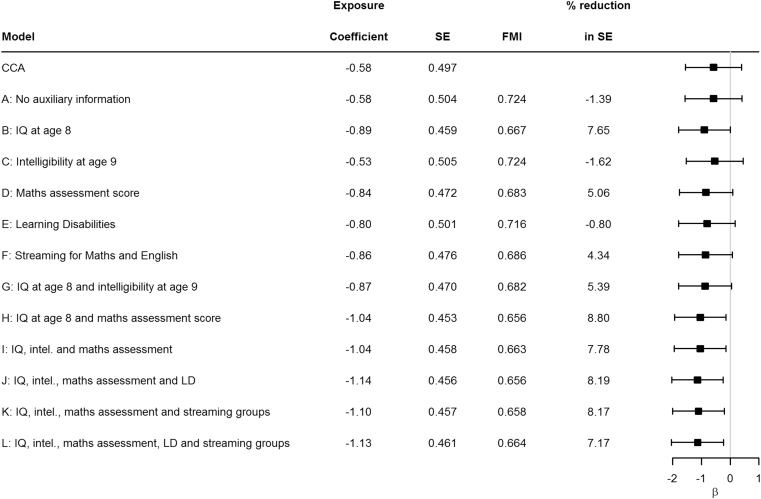
Estimate, standard error, and FMI for the exposure coefficient in the applied example adjusted analysis model. Reduction in SE is relative to CCA. CCA = complete case analysis; FMI = fraction of missing information; SE = standard error.

The results for the estimate, SE, FMI, and percentage reduction in SE compared with CCA for the exposure coefficient of the adjusted linear regression are presented in Figure 3. The estimated association between maternal smoking and IQ is further from the null when the imputation model includes more variables. The estimates provided by the CCA model would lead to different conclusions to those provided by MI models H–L.

Figure 3 shows that for the exposure coefficient, the MI SEs for most imputation models were smaller than that of CCA; models A, C, and E are exceptions displaying slight increases, likely because of these models containing low levels of auxiliary information. No model led to larger FMI than that of model A, which included no auxiliary information.

Including more than one auxiliary variable in the imputation model had inconsistent influence on FMI and SE for the exposure coefficient. For example, the addition of intelligibility to model B (see model G) led to increased FMI and a reduced gain in efficiency versus CCA, as measured by percentage reduction in SE. The addition of the maths assessment score to model B (see model H) led to the greatest estimate precision and lowest FMI. Once intelligibility had been added to model H (see models I–L), further addition of variables to the model could not achieve the efficiency gains observed in model H. It is possible that this is because missing information in intelligibility led to increased variability that could not be counteracted by introducing further information about missing outcomes via the inclusion of more auxiliary variables. The confidence intervals of the exposure coefficient estimates overlap for all imputation models investigated.

Comparison of Figure 3 with Supplementary Figure S3 shows that greater reductions in efficiency, relative to CCA, were made when the analysis model was an unadjusted model. This is because confounders are likely to explain some of the covariation between the exposure and outcome as well as some of the missingness in the outcome. The remaining unexplained variation that is available to be accounted for by auxiliary variables is therefore less in the adjusted models.

## 4 Discussion

Our study showed that at all proportions of missingness in the outcome, there is benefit to using MI in terms of reducing bias and improving efficiency and that FMI can be used as a better guide to the efficiency gains to be made from MI than the proportion of missing data. We found that, compared to CCA, MI with auxiliary information improved efficiency of effect estimates at any proportion of missing data. Provided the imputation model was correctly specified and included all variables related to missingness then MI eliminated bias when data were MAR regardless of the amount of missing data. CCA was always biased because the analysis model did not include all variables related to missingness [6], [28], [38]. Our simulations (both MCAR and MAR) revealed that similar FMI values can result from data sets with differing proportions of missing data if they have differing amounts of auxiliary information. In models with the same FMI, the empirical SE was approximately equal despite the different proportions of missing data. The biggest factor affecting the gain in precision of effect estimates from using MI is therefore not the proportion of missing data but instead the FMI.

The results of the applied example show that auxiliary information influences the SE and FMI of effect estimates in a real-world data set. The example also demonstrates that the introduction of extra variables to the imputation model, without reducing the FMI, can be harmful to the precision of model estimates. This can likely be explained by the additional missing data in the auxiliary variable leading to a loss in estimate precision. Of all models tested, we would recommend the use of model J because it had the lowest FMI and included more variables that predicted missingness than model H which had an equivalent FMI. Model L additionally included the streaming group variables, which also predicted missingness, but there was very little difference in the coefficient estimate compared with model J although its FMI was greater than model J.

An inclusive strategy of auxiliary variables has been suggested as preferable to a restrictive strategy to try to include all variables that may be associated with the missingness mechanism [39]. Using too many auxiliary variables is harmful, however, when the sample size is small [40]. This leads to a ratio of observed values to model parameters that is close to unity which in turn leads to poor model fit. Where the sample size is large, an inclusive strategy of auxiliary variables is acceptable; however, our results show that the FMI should be checked to see whether missing data in auxiliary variables decreased efficiency (as in our applied example). Those variables which make the MAR assumption plausible should always be included in the imputation model.

Our simulation study was limited by its single sample size, simple analysis model, and that we considered missingness in only one variable. In real-world data sets, auxiliary variables are often correlated, which will reduce the independent contribution of each variable to the imputation model but may aid in prediction of missing values in an auxiliary variable itself. Missingness often occurs in several variables within a data set, although this should not bias the estimate of the effect of exposure on outcome, provided missingness is not related to the outcome (for CCA) or that all variables are MAR (for MI) [28]. Sample size has been shown to influence efficiency gains obtained via MI for binary outcomes [25] with smaller sample sizes associated with smaller gains at equivalent proportions of missing data. It is possible that greater efficiency gains could be achieved at the smaller proportions of missing data than was observed in our study if a greater sample size was used. Bias reduction has also been found to be greater with increasing sample size for longitudinal data [22]. Finally, we have only investigated correctly specified MI—if the imputation model is incorrectly specified, the bias may not be completely removed or could even be larger than in the CCA [9], [10], [41]. In practice, the variables related to missingness are seldom known with certainty.

Further work needs to investigate the applicability of our results to models with binary and time-to-event outcomes. Logistic regression sometimes differs to linear regression with regard to missing data; for example, logistic regression is more robust to bias in the presence of missing data [42]. In the supplementary material, we display a simple example of our simulation study for a binary outcome. For MI of a logistic regression analysis model, the simulation results show that the FMI is reduced with increasing auxiliary information, which was also shown by the results of our simulation study for the linear regression model. More thorough investigation is warranted.

Our study is the first to investigate the influence of increasing auxiliary information on bias and efficiency of MI analyses at proportions of missing data greater than 50% missingness. Studies that have looked at large proportions of missing data, in the absence of auxiliary information, have also shown MI to reduce bias and improve efficiency over CCA [23], [24]. These studies highlighted the importance of a properly specified imputation model to reducing bias.

For MI to be valid, the data must be MAR (given the variables in the imputation model) and both analysis and imputation models must be correctly specified. This may be harder to investigate as the number of participants with complete data (rather than the proportion of the sample with missing data) decreases. For example, investigating whether interactions or nonlinearities need to be included in the imputation model will be harder as the number of complete cases gets smaller. However, the CCA also depends on the analysis model being correctly specified and data being MAR, given the variables in the analysis model. These assumptions will be similarly hard to investigate as the number of complete cases decreases. Thus, where conclusions are being drawn from a small number of complete cases, we recommend sensitivity analyses to explore a range of plausible analysis and imputation models, as well as the impact of deviations from MAR [9], [43].

Our results have important implications for epidemiologists, and reviewers, for the conduct and reporting of analysis on incomplete data. Our results imply that researchers should consider whether all the variables related to missingness can plausibly be included in the imputation model (to limit bias), and then whether there are auxiliary variables that can lower the FMI (to improve efficiency). We recommend that all articles reporting results of analyses with incomplete data show a table of characteristics of those with complete data vs those with incomplete data (to assess factors associated with missingness) and a table showing variables associated with incomplete variables (to assess auxiliary information). The FMI of MI analyses should be reported, along with a discussion of whether it is plausible that all variables related to missingness have been included in the imputation models.

A key finding of this study is that the proportion of missing data should not be used as a guide to whether to use MI (or CCA) or not—we have shown that correctly specified MI can reduce bias and improve efficiency for analysis of MAR data at any proportion of missingness. If we cannot correctly specify the imputation model, then alternatives to MI such as inverse probability weighting [44] or study-specific sensitivity analysis may be preferable. Our work shows that the FMI provides better insight into the amount of information retained using MI than does the proportion of missing data. It may be useful to check the FMI when adding auxiliary variables to an imputation model to see which variables are not adding information (e.g., due to the proportion of missing data in an auxiliary variable).

## Appendix A Supplementary data

Data Profile
