# Supplementary material for: The proportion of missing data should not be used to guide decisions on multiple imputation
Source: J Clin Epidemiol. 2019 Jun;110:63–73. doi: 10.1016/j.jclinepi.2019.02.016 (PMC6547017; doi:10.1016/j.jclinepi.2019.02.016)
Supplement: Supplement [file mmc2.docx]

SUPPLEMENTARY MATERIAL

Contents

[The fraction of missing information (FMI) 2](#_Toc536801781)

[Calculation of performance statistics for the simulation study 3](#_Toc536801782)

[Justification for the number of imputations used 5](#_Toc536801783)

[Empirical SE of the MI exposure coefficient against FMI - Figure 1 separated by panels of percentage missing data 6](#_Toc536801784)

[Comparison to CCA for bias and SE of the constant coefficient of MCAR and MAR data in the simulation study 7](#_Toc536801785)

[Performance statistics for the exposure coefficient in the simulation study 9](#_Toc536801786)

[Performance statistics for the constant coefficient in the simulation study 13](#_Toc536801787)

[FMI and efficiency gains were not sensitive to whether the auxiliary variable was included in the missingness mechanism 17](#_Toc536801788)

[Missing data in included and excluded sample for the applied example 19](#_Toc536801789)

[Missing data pattern in the applied example 19](#_Toc536801790)

[Applied example exposure coefficient results for the unadjusted model 20](#_Toc536801791)

[Applied example constant coefficient results 21](#_Toc536801792)

[Simulation study using a binary outcome 22](#_Toc536801793)

# The fraction of missing information (FMI)

For an estimate $\hat{\beta}$, which could be a regression coefficient that has been averaged across $m$ imputed datasets (i.e. $\hat{\beta}=(1/m\boldsymbol{)}\sum_{\boldsymbol{k=1}}^{\boldsymbol{m}} \hat{\beta}_{k}$) the FMI is given by

$$FMI\boldsymbol{=}\frac{\boldsymbol{B}}{\boldsymbol{(W+B)}}.$$

Here $\boldsymbol{W}$ is the within imputation variance of $\hat{\beta}$ and$\boldsymbol{B}$ is the between imputation variance of$\hat{\beta}$. These values are derived as follows. For$\hat{V}_{k}$, the squared standard error of$\hat{\beta}$in the$k^{th}$ imputed dataset, the within imputation variance across $m$imputations is given by

$$\boldsymbol{W}=(1/m\boldsymbol{)}\sum_{k=1}^{m} \hat{V}_{k}.$$

In other words, $\boldsymbol{W}$ is calculated as the average squared standard error of $\hat{\beta}$. The between imputation variance is given by

$$\boldsymbol{B=}\left( \frac{1}{m-1} \right)\sum_{k=1}^{m} (\hat{\beta}_{k}- \hat{\beta})^{2}.$$

This is the square of the standard deviation of the estimated regression coefficient in each imputed dataset relative to the mean value across all imputed datasets.

The total variance,$var(\hat{\beta})$ is equal to$\boldsymbol{W}+\left( 1+\frac{1}{m} \right)\boldsymbol{B}$. Hence for large numbers of imputations$m$*,* the FMI is the fraction of the total variance that is attributable to between imputation variance.

Being a fraction of the total variance, the FMI can take values between 0 and 1. Low values (i.e. close to 0) indicate that much of the “missing” information is in fact captured by other, more completely observed, variables. In the absence of any missing data the FMI is 0 because the between imputation variance,$\boldsymbol{B,}$ would be 0 since all imputation datasets would be identical. Of course, if we have no missing data we would not use multiple imputation and therefore would not estimate the FMI.

# Calculation of performance statistics for the simulation study

1. **Bias**

Bias is calculated as the difference between the estimated and true values of$\beta_{j}$,

$$Bias=\hat{\beta}_{j}-\beta_{j},$$

where the true values are given by

$$\beta_{1}= \frac{Cov(Y,X)}{Var(X)} ,$$

$$\beta_{0}=\bar{y}{-\beta}_{1}\bar{x}.$$

Here $\bar{y}$ and $\bar{x}$ are the arithmetic means of the variables *Y* (outcome) and *X* (exposure) respectively.

1. **Empirical standard error**

Forsimulations $q=\left( 1,\ldots,r \right)$, $\hat{\beta}_{q}$ equal to the estimate averaged across imputations in simulation $q$, and $\bar{\beta}=\left( 1/r \right)\sum_{q=1}^{r} \hat{\beta}_{q}$, the empirical standard deviation is given by

$$\mathrm{emp}. s.e\boldsymbol{.=}\sqrt{\left( \frac{1}{r-1} \right)\sum_{q}^{r} \left( \hat{\beta}_{q}- \bar{\beta} \right)^{2}}.$$

Note the difference between empirical standard deviation and between imputation variance is that the former is calculated across simulations while the latter is calculated across imputations within a single simulation.

1. **Average estimated standard errors**

For$s_{q}$, the standard error of$\hat{\beta}$in the $q^{th}$ simulation, the average estimated standard error is given by

$$model s.e.=\sqrt{\bar{s^{2}}}=\sqrt{\frac{1}{r}\sum_{q=1}^{r} s_{q}^{2}}$$

We do not present the average standard errors. These are used to calculate the relative percentage error.

1. **Relative percentage error**

The relative percentage error is the proportional error of the average estimated standard errors$\sqrt{\bar{s^{2}}}$, relative to the empirical standard deviation of $\hat{\beta}_{j}.$

$$Relative \% Error= \left( \frac{model s.e.}{emp. s.e.}\boldsymbol{-}1 \right)\times100$$

The empirical standard error and the average estimated standard error should be approximately equal. The relative percentage error is therefore a measure of bias in $s_{j}$ with departures from 0 indicating bias. Relative percentage error was calculated using the *simsum* command in Stata.

1. **Coverage probability of 95% CI**

Letting $I(.)$ be the indicator function, the coverage of a 95% confidence interval is given by

$$C=\frac{1}{r}\sum_{q=1}^{r} I( \left| \hat{\beta}_{q}-\beta\right|< z_{\alpha/2}s_{q} ),$$

where $z_{\alpha/2}=1.96$is the critical value of the normal distribution. The coverage probability indicates the percentage of simulations in which the true value of the coefficient is found within the span of the estimated 95% confidence interval. A value greater than 95% indicates over-coverage while a value less than 95% indicates under-coverage.

# Justification for the number of imputations used

Figure S1 displays the FMI of the $\hat{\beta}_{1}$ coefficient for 20%, 40%, 60% and 80% missing data for increasing numbers of imputations. 47 simulations, all displayed, were performed. An imputation model including Y, X, Z_1_ and Z_3_ was used along with the same analysis model used ($Y$ regressed on $X$) as the main investigation. The datasets used were also the same as those used in the main investigation. Multiple imputation was performed using the Stata package *mi impute.* As Stata allows a maximum of 1000 imputations only the FMI had to be calculated manually instead of being obtained directly from Stata’s *mi estimate*. The analysis model was performed on each imputed dataset using Stata’s *regress* command. Following this the $\hat{\beta}_{1}$ coefficient for each imputed dataset was appended together and the FMI based on the first $m$ imputations was calculated. FMI was calculated at every 5 imputations from 5-50 imputations, every 25 imputations from 25 to 500 imputations and every 100 imputations from 500 to 10000 imputations.

The FMI is highly variable at low numbers of imputations, only becoming stable for all proportions of missing data when 250 imputations are used. At 1000 imputations we therefore have greater confidence that the FMI estimates across simulations have become stable. The figure also seems to show that the FMI is more variable with greater proportions of missing data at lower numbers of imputations.


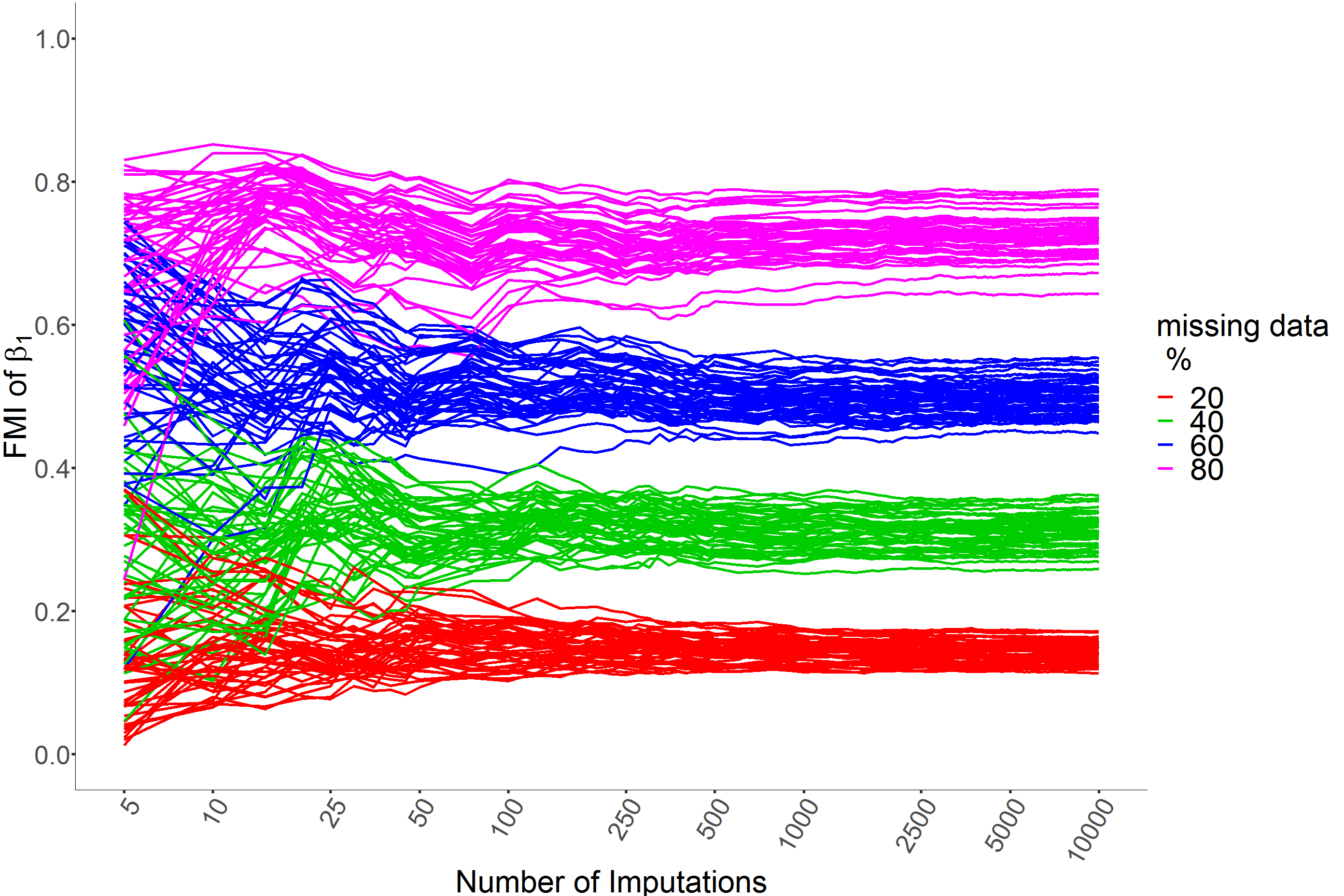


Figure S1. Line graph of FMI for regression coefficient of $\boldsymbol{Y}$ on $\boldsymbol{X}$ against the number of imputations.

# Empirical SE of the MI exposure coefficient against FMI - Figure 1 separated by panels of percentage missing data


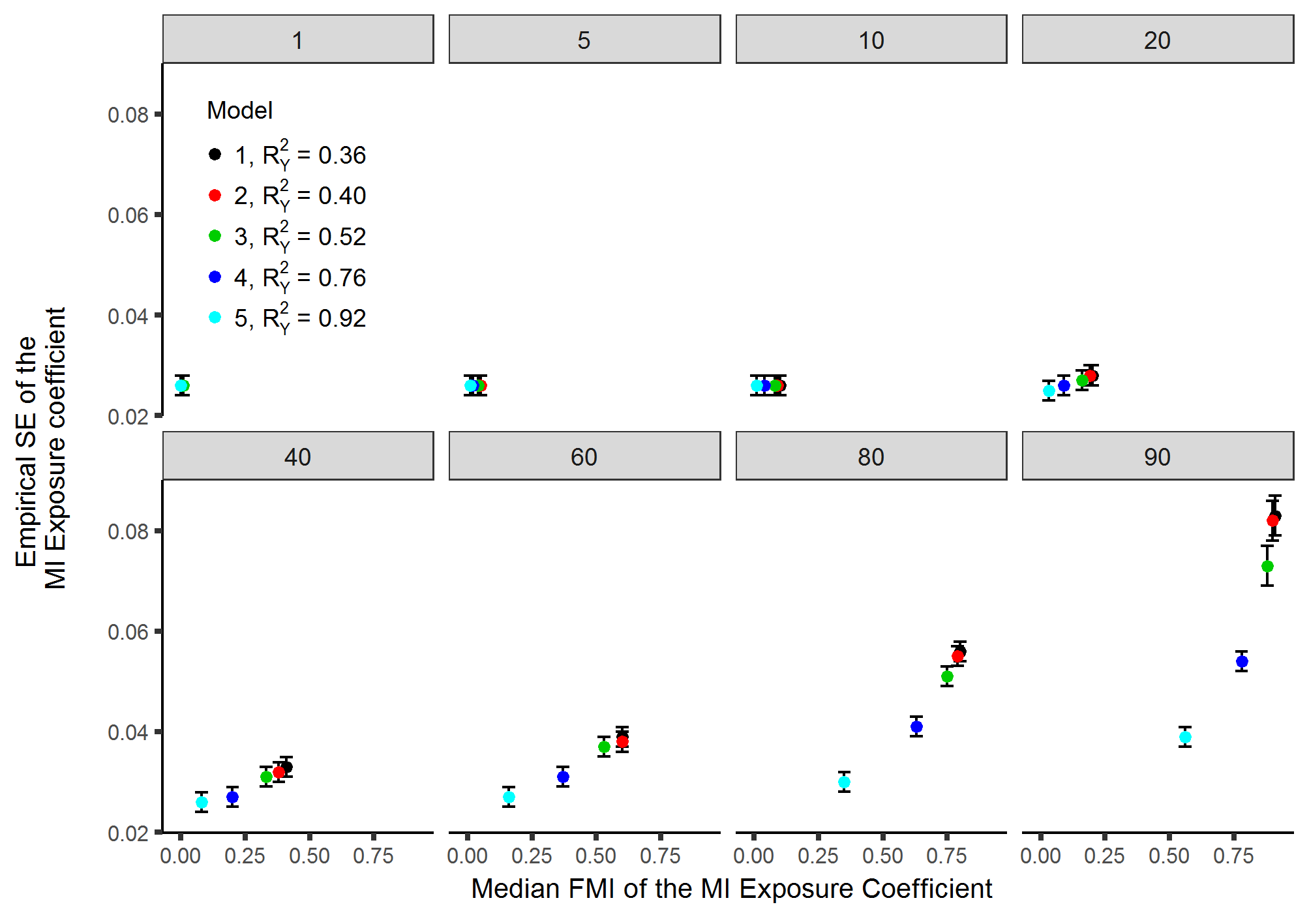


Figure S2. Empirical SE of the MI exposure coefficient plotted against FMI for simulated MCAR data. Panels separate the data by percentage of missing data. Error bars are 95% confidence intervals based on Monte Carlo standard errors across simulations. FMI = fraction of missing information; SE = standard error.

# Comparison to CCA for bias and SE of the constant coefficient of MCAR and MAR data in the simulation study

Table S1. Percentage Reduction in Empirical SE and Bias Compared to CCA for MCAR and MAR Results of the Constant Coefficient in the Simulation Study.

|  |  | % Reduction in SE compared to CCA ^c^ | | % Reduction in bias compared to CCA ^d^ |
| --- | --- | --- | --- | --- |
| % Missing | Imputation model ^a b^ | MCAR SE | MAR SE | MAR Bias |
| 1 | 1: R^2^=0.36 (No aux info) | 0.00% | -0.02% | 2.53% |
|  | 2: R^2^=0.40 | 0.04% | -0.16% | 1.88% |
|  | 3: R^2^=0.52 | 0.29% | 0.15% | 73.00% |
|  | 4: R^2^=0.76 | 0.29% | 0.22% | 74.05% |
|  | 5: R^2^=0.92 | 0.51% | 0.17% | 71.75% |
| 5 | 1: R^2^=0.36 (No aux info) | 0.00% | 0.01% | 0.13% |
|  | 2: R^2^=0.40 | -0.15% | 0.03% | -0.44% |
|  | 3: R^2^=0.52 | 0.72% | -0.28% | 93.30% |
|  | 4: R^2^=0.76 | 1.37% | 0.52% | 94.75% |
|  | 5: R^2^=0.92 | 1.90% | 0.71% | 95.05% |
| 10 | 1: R^2^=0.36 (No aux info) | 0.00% | 0.00% | 0.40% |
|  | 2: R^2^=0.40 | -0.08% | 0.80% | 0.13% |
|  | 3: R^2^=0.52 | 1.26% | 0.32% | 95.98% |
|  | 4: R^2^=0.76 | 2.92% | 2.66% | 96.52% |
|  | 5: R^2^=0.92 | 3.69% | 4.34% | 97.13% |
| 20 | 1: R^2^=0.36 (No aux info) | 0.00% | -0.01% | -0.17% |
|  | 2: R^2^=0.40 | 0.43% | 0.48% | -0.75% |
|  | 3: R^2^=0.52 | 3.40% | -0.26% | 99.31% |
|  | 4: R^2^=0.76 | 6.67% | 5.68% | 99.29% |
|  | 5: R^2^=0.92 | 7.56% | 8.67% | 99.10% |
| 40 | 1: R^2^=0.36 (No aux info) | 0.00% | -0.10% | -0.23% |
|  | 2: R^2^=0.40 | 0.73% | 0.97% | 0.32% |
|  | 3: R^2^=0.52 | 5.49% | -0.65% | 99.60% |
|  | 4: R^2^=0.76 | 13.24% | 11.50% | 99.15% |
|  | 5: R^2^=0.92 | 17.72% | 19.20% | 99.22% |
| 60 | 1: R^2^=0.36 (No aux info) | 0.00% | -0.15% | 0.22% |
|  | 2: R^2^=0.40 | 1.99% | 2.61% | 0.51% |
|  | 3: R^2^=0.52 | 6.00% | -0.98% | 99.79% |
|  | 4: R^2^=0.76 | 20.37% | 16.06% | 99.20% |
|  | 5: R^2^=0.92 | 29.46% | 34.27% | 99.57% |
| 80 | 1: R^2^=0.36 (No aux info) | 0.00% | -0.07% | -0.08% |
|  | 2: R^2^=0.40 | 1.93% | 2.66% | 0.31% |
|  | 3: R^2^=0.52 | 9.93% | -5.45% | 99.86% |
|  | 4: R^2^=0.76 | 28.05% | 19.48% | 99.36% |
|  | 5: R^2^=0.92 | 44.15% | 43.23% | 99.70% |
| 90 | 1: R^2^=0.36 (No aux info) | -0.01% | 0.03% | 0.19% |
|  | 2: R^2^=0.40 | 2.79% | 2.29% | 0.62% |
|  | 3: R^2^=0.52 | 11.55% | -11.73% | 99.25% |
|  | 4: R^2^=0.76 | 32.45% | 16.95% | 99.07% |
|  | 5: R^2^=0.92 | 52.57% | 45.83% | 98.98% |

CCA – Complete case analysis; MAR – Missing at random; MCAR – Missing completely at random; SE – Standard error

^a^ R^2^ refers to the squared coefficient of multiple correlation which is used as a measure of auxiliary information

^b^ Models 1 and 2 do not include all variables in the missingness mechanism and so are biased (as expected) for the MAR data. Models 3-5 do include all variables in the missingness mechanism and so are unbiased (as expected).

^c^ Calculated using 100 × (se_CCA_ – se_MI_)/se_CCA_ where se_CCA_ and se_MI_ are the empirical standard error of the CCA model and the MI model respectively

^d^ Calculated using 100 × (abs(bias_CCA_)-abs(bias_MI_))/abs(bias_CCA_) where abs(.) is a function giving the absolute value and bias_CCA_ and bias_MI_ are the bias of the CCA model and the MI model respectively.

# Performance statistics for the exposure coefficient in the simulation study

Table S2. Performance Statistics for the MCAR Results of the Exposure Coefficient in the Simulation Study.

| Statistic | % missing data | CCA | | Model 1  No aux info, R^2^=0.36 | | Model 2  R^2^=0.40 | | Model 3  R^2^=0.52 | | Model 4  R^2^=0.76 | | Model 5  R^2^=0.92 | |
| --- | --- | --- | --- | --- | --- | --- | --- | --- | --- | --- | --- | --- | --- |
| FMI ^a^ | 1 |  |  | 0.01 | 0.01, 0.01 | 0.01 | 0.01, 0.01 | 0.01 | 0.00, 0.01 | 0.00 | 0.00, 0.00 | 0.00 | 0.00, 0.00 |
|  | 5 |  |  | 0.05 | 0.04, 0.06 | 0.05 | 0.04, 0.05 | 0.04 | 0.03, 0.04 | 0.02 | 0.02, 0.02 | 0.01 | 0.01, 0.01 |
|  | 10 |  |  | 0.10 | 0.09, 0.11 | 0.09 | 0.08, 0.10 | 0.08 | 0.07, 0.08 | 0.04 | 0.04, 0.05 | 0.01 | 0.01, 0.02 |
|  | 20 |  |  | 0.20 | 0.19, 0.21 | 0.19 | 0.18, 0.20 | 0.16 | 0.15, 0.17 | 0.09 | 0.08, 0.09 | 0.03 | 0.03, 0.03 |
|  | 40 |  |  | 0.41 | 0.39, 0.42 | 0.38 | 0.37, 0.40 | 0.33 | 0.32, 0.35 | 0.20 | 0.19, 0.21 | 0.08 | 0.07, 0.08 |
|  | 60 |  |  | 0.60 | 0.59, 0.62 | 0.60 | 0.58, 0.61 | 0.53 | 0.52, 0.55 | 0.37 | 0.35, 0.39 | 0.16 | 0.15, 0.17 |
|  | 80 |  |  | 0.80 | 0.79, 0.81 | 0.79 | 0.78, 0.81 | 0.75 | 0.73, 0.76 | 0.63 | 0.60, 0.65 | 0.35 | 0.33, 0.38 |
|  | 90 |  |  | 0.91 | 0.90, 0.91 | 0.90 | 0.89, 0.91 | 0.88 | 0.86, 0.89 | 0.78 | 0.76, 0.80 | 0.56 | 0.53, 0.60 |
|  |  |  |  |  |  |  |  |  |  |  |  |  |  |
| Bias | 1 | 0.00 | 0.00, 0.00 | 0.00 | 0.00, 0.00 | 0.00 | 0.00, 0.00 | 0.00 | 0.00, 0.00 | 0.00 | 0.00, 0.00 | 0.00 | 0.00, 0.00 |
|  | 5 | 0.00 | 0.00, 0.00 | 0.00 | 0.00, 0.00 | 0.00 | 0.00, 0.00 | 0.00 | 0.00, 0.00 | 0.00 | 0.00, 0.00 | 0.00 | 0.00, 0.00 |
|  | 10 | 0.00 | 0.00, 0.00 | 0.00 | 0.00, 0.00 | 0.00 | 0.00, 0.00 | 0.00 | 0.00, 0.00 | 0.00 | 0.00, 0.00 | 0.00 | 0.00, 0.00 |
|  | 20 | 0.00 | 0.00, 0.00 | 0.00 | 0.00, 0.00 | 0.00 | 0.00, 0.00 | 0.00 | 0.00, 0.00 | 0.00 | 0.00, 0.00 | 0.00 | 0.00, 0.00 |
|  | 40 | 0.00 | 0.00, 0.00 | 0.00 | 0.00, 0.00 | 0.00 | 0.00, 0.00 | 0.00 | 0.00, 0.00 | 0.00 | 0.00, 0.00 | 0.00 | 0.00, 0.00 |
|  | 60 | 0.00 | 0.00, 0.00 | 0.00 | 0.00, 0.00 | 0.00 | 0.00, 0.00 | 0.00 | 0.00, 0.00 | 0.00 | 0.00, 0.00 | 0.00 | 0.00, 0.00 |
|  | 80 | 0.00 | 0.00, 0.00 | 0.00 | 0.00, 0.00 | 0.00 | -0.01, 0.00 | 0.00 | 0.00, 0.00 | 0.00 | 0.00, 0.00 | 0.00 | 0.00, 0.00 |
|  | 90 | 0.00 | 0.00, 0.01 | 0.00 | 0.00, 0.01 | 0.00 | 0.00, 0.01 | 0.00 | 0.00, 0.01 | 0.00 | 0.00, 0.00 | 0.00 | 0.00, 0.00 |
|  |  |  |  |  |  |  |  |  |  |  |  |  |  |
| Empirical SE | 1 | 0.03 | 0.02, 0.03 | 0.03 | 0.02, 0.03 | 0.03 | 0.02, 0.03 | 0.03 | 0.02, 0.03 | 0.03 | 0.02, 0.03 | 0.03 | 0.02, 0.03 |
|  | 5 | 0.03 | 0.02, 0.03 | 0.03 | 0.02, 0.03 | 0.03 | 0.02, 0.03 | 0.03 | 0.02, 0.03 | 0.03 | 0.02, 0.03 | 0.03 | 0.02, 0.03 |
|  | 10 | 0.03 | 0.03, 0.03 | 0.03 | 0.03, 0.03 | 0.03 | 0.03, 0.03 | 0.03 | 0.03, 0.03 | 0.03 | 0.02, 0.03 | 0.03 | 0.02, 0.03 |
|  | 20 | 0.03 | 0.03, 0.03 | 0.03 | 0.03, 0.03 | 0.03 | 0.03, 0.03 | 0.03 | 0.03, 0.03 | 0.03 | 0.02, 0.03 | 0.03 | 0.02, 0.03 |
|  | 40 | 0.03 | 0.03, 0.03 | 0.03 | 0.03, 0.03 | 0.03 | 0.03, 0.03 | 0.03 | 0.03, 0.03 | 0.03 | 0.03, 0.03 | 0.03 | 0.02, 0.03 |
|  | 60 | 0.04 | 0.04, 0.04 | 0.04 | 0.04, 0.04 | 0.04 | 0.04, 0.04 | 0.04 | 0.04, 0.04 | 0.03 | 0.03, 0.03 | 0.03 | 0.03, 0.03 |
|  | 80 | 0.06 | 0.05, 0.06 | 0.06 | 0.05, 0.06 | 0.05 | 0.05, 0.06 | 0.05 | 0.05, 0.05 | 0.04 | 0.04, 0.04 | 0.03 | 0.03, 0.03 |
|  | 90 | 0.08 | 0.08, 0.09 | 0.08 | 0.08, 0.09 | 0.08 | 0.08, 0.09 | 0.07 | 0.07, 0.08 | 0.05 | 0.05, 0.06 | 0.04 | 0.04, 0.04 |
|  |  |  |  |  |  |  |  |  |  |  |  |  |  |
| Relative error | 1 | -1.34 | -5.67, 3.00 | -1.36 | -5.69, 2.97 | -1.19 | -5.52, 3.15 | -1.26 | -5.59, 3.07 | -1.10 | -5.44, 3.24 | -1.24 | -5.58, 3.09 |
|  | 5 | -0.53 | -4.90, 3.84 | -0.52 | -4.89, 3.85 | -0.49 | -4.86, 3.87 | -0.10 | -4.48, 4.29 | -0.08 | -4.46, 4.31 | -1.14 | -5.48, 3.19 |
|  | 10 | 0.96 | -3.47, 5.39 | 0.89 | -3.54, 5.32 | 1.03 | -3.41, 5.46 | 0.30 | -4.10, 4.70 | 0.44 | -3.97, 4.85 | -0.62 | -4.98, 3.74 |
|  | 20 | 0.92 | -3.51, 5.35 | 0.77 | -3.65, 5.20 | 1.47 | -2.98, 5.93 | 1.03 | -3.41, 5.47 | 2.93 | -1.58, 7.45 | 2.47 | -2.02, 6.97 |
|  | 40 | 0.63 | -3.79, 5.05 | 1.09 | -3.35, 5.53 | 1.46 | -2.99, 5.92 | 1.04 | -3.40, 5.48 | 3.25 | -1.28, 7.78 | 2.94 | -1.58, 7.46 |
|  | 60 | 2.37 | -2.13, 6.87 | 2.81 | -1.71, 7.33 | 4.80 | 0.19, 9.40 | 0.42 | -3.99, 4.84 | 3.28 | -1.25, 7.82 | 3.47 | -1.07, 8.02 |
|  | 80 | 1.80 | -2.69, 6.29 | 1.11 | -3.35, 5.57 | 2.56 | -1.96, 7.08 | -1.52 | -5.85, 2.82 | 3.14 | -1.40, 7.68 | 4.61 | 0.01, 9.20 |
|  | 90 | -2.06 | -6.40, 2.28 | -0.47 | -4.88, 3.93 | -0.71 | -5.11, 3.68 | -0.10 | -4.52, 4.32 | 1.21 | -3.26, 5.69 | 0.38 | -4.05, 4.80 |
|  |  |  |  |  |  |  |  |  |  |  |  |  |  |
| Coverage | 1 | 94.70 | 93.31, 96.09 | 94.60 | 93.20, 96.00 | 94.60 | 93.20, 96.00 | 94.90 | 93.54, 96.26 | 94.70 | 93.31, 96.09 | 94.90 | 93.54, 96.26 |
|  | 5 | 95.20 | 93.88, 96.52 | 95.30 | 93.99, 96.61 | 95.20 | 93.88, 96.52 | 95.40 | 94.10, 96.70 | 95.00 | 93.65, 96.35 | 94.70 | 93.31, 96.09 |
|  | 10 | 95.50 | 94.22, 96.78 | 95.40 | 94.10, 96.70 | 95.50 | 94.22, 96.78 | 95.10 | 93.76, 96.44 | 95.30 | 93.99, 96.61 | 94.90 | 93.54, 96.26 |
|  | 20 | 94.70 | 93.31, 96.09 | 94.80 | 93.42, 96.18 | 94.80 | 93.42, 96.18 | 94.40 | 92.97, 95.83 | 95.50 | 94.22, 96.78 | 95.50 | 94.22, 96.78 |
|  | 40 | 94.90 | 93.54, 96.26 | 94.90 | 93.54, 96.26 | 94.90 | 93.54, 96.26 | 94.40 | 92.97, 95.83 | 95.20 | 93.88, 96.52 | 95.80 | 94.56, 97.04 |
|  | 60 | 96.20 | 95.01, 97.39 | 96.20 | 95.01, 97.39 | 96.30 | 95.13, 97.47 | 95.00 | 93.65, 96.35 | 95.50 | 94.22, 96.78 | 95.30 | 93.99, 96.61 |
|  | 80 | 95.00 | 93.65, 96.35 | 94.70 | 93.31, 96.09 | 95.70 | 94.44, 96.96 | 93.90 | 92.42, 95.38 | 95.50 | 94.22, 96.78 | 95.90 | 94.67, 97.13 |
|  | 90 | 94.90 | 93.54, 96.26 | 95.30 | 93.99, 96.61 | 94.90 | 93.54, 96.26 | 94.30 | 92.86, 95.74 | 95.30 | 93.99, 96.61 | 95.10 | 93.76, 96.44 |

CCA – Complete case analysis; FMI- Fraction of Missing Information; SE – Standard error; R^2^ – the squared coefficient of multiple correlation

^a^ For FMI the estimate was the median across simulations and the interval represents the interquartile range. For all other statistics the mean across simulations was taken and the 95% confidence interval was calculated using Monte Carlo standard error.

Table S3. Performance Statistics for the MAR Results of the Exposure Coefficient in the Simulation Study.

| Statistic | % missing data | CCA | | Model 1  No aux info, R^2^=0.36 | | Model 2  R^2^=0.40 | | Model 3  R^2^=0.52 | | Model 4  R^2^=0.76 | | Model 5  R^2^=0.92 | |
| --- | --- | --- | --- | --- | --- | --- | --- | --- | --- | --- | --- | --- | --- |
| FMI ^a^ | 1 |  |  | 0.02 | 0.01, 0.02 | 0.02 | 0.01, 0.02 | 0.01 | 0.01, 0.02 | 0.01 | 0.00, 0.01 | 0.00 | 0.00, 0.00 |
|  | 5 |  |  | 0.08 | 0.07, 0.09 | 0.08 | 0.07, 0.09 | 0.06 | 0.05, 0.07 | 0.03 | 0.03, 0.04 | 0.01 | 0.01, 0.01 |
|  | 10 |  |  | 0.15 | 0.14, 0.17 | 0.15 | 0.13, 0.16 | 0.12 | 0.11, 0.14 | 0.06 | 0.06, 0.07 | 0.02 | 0.02, 0.03 |
|  | 20 |  |  | 0.27 | 0.25, 0.29 | 0.26 | 0.24, 0.28 | 0.22 | 0.21, 0.24 | 0.13 | 0.11, 0.14 | 0.05 | 0.04, 0.05 |
|  | 40 |  |  | 0.48 | 0.45, 0.49 | 0.46 | 0.44, 0.48 | 0.42 | 0.40, 0.44 | 0.26 | 0.25, 0.28 | 0.11 | 0.10, 0.12 |
|  | 60 |  |  | 0.66 | 0.64, 0.68 | 0.64 | 0.62, 0.66 | 0.60 | 0.58, 0.62 | 0.43 | 0.41, 0.45 | 0.20 | 0.19, 0.22 |
|  | 80 |  |  | 0.83 | 0.81, 0.84 | 0.82 | 0.80, 0.84 | 0.79 | 0.77, 0.81 | 0.65 | 0.63, 0.68 | 0.39 | 0.36, 0.43 |
|  | 90 |  |  | 0.91 | 0.90, 0.92 | 0.91 | 0.90, 0.92 | 0.89 | 0.87, 0.90 | 0.80 | 0.78, 0.82 | 0.59 | 0.55, 0.63 |
|  |  |  |  |  |  |  |  |  |  |  |  |  |  |
| Bias | 1 | 0.00 | -0.01, 0.00 | 0.00 | -0.01, 0.00 | 0.00 | -0.01, 0.00 | 0.00 | 0.00, 0.00 | 0.00 | 0.00, 0.00 | 0.00 | 0.00, 0.00 |
|  | 5 | -0.01 | -0.02, -0.01 | -0.01 | -0.02, -0.01 | -0.01 | -0.02, -0.01 | 0.00 | 0.00, 0.00 | 0.00 | 0.00, 0.00 | 0.00 | 0.00, 0.00 |
|  | 10 | -0.02 | -0.03, -0.02 | -0.02 | -0.03, -0.02 | -0.02 | -0.03, -0.02 | 0.00 | 0.00, 0.00 | 0.00 | 0.00, 0.00 | 0.00 | 0.00, 0.00 |
|  | 20 | -0.04 | -0.04, -0.04 | -0.04 | -0.04, -0.04 | -0.04 | -0.04, -0.04 | 0.00 | 0.00, 0.00 | 0.00 | 0.00, 0.00 | 0.00 | 0.00, 0.00 |
|  | 40 | -0.06 | -0.06, -0.06 | -0.06 | -0.06, -0.06 | -0.06 | -0.06, -0.05 | 0.00 | 0.00, 0.00 | 0.00 | 0.00, 0.00 | 0.00 | 0.00, 0.00 |
|  | 60 | -0.06 | -0.07, -0.06 | -0.06 | -0.07, -0.06 | -0.06 | -0.07, -0.06 | 0.00 | 0.00, 0.00 | 0.00 | 0.00, 0.00 | 0.00 | 0.00, 0.00 |
|  | 80 | -0.06 | -0.07, -0.06 | -0.06 | -0.07, -0.06 | -0.06 | -0.07, -0.06 | 0.00 | -0.01, 0.00 | 0.00 | 0.00, 0.00 | 0.00 | 0.00, 0.00 |
|  | 90 | -0.05 | -0.06, -0.05 | -0.05 | -0.06, -0.05 | -0.05 | -0.06, -0.05 | 0.00 | 0.00, 0.00 | 0.00 | 0.00, 0.01 | 0.00 | 0.00, 0.00 |
|  |  |  |  |  |  |  |  |  |  |  |  |  |  |
| Empirical SE | 1 | 0.02 | 0.02, 0.03 | 0.02 | 0.02, 0.03 | 0.02 | 0.02, 0.03 | 0.02 | 0.02, 0.03 | 0.02 | 0.02, 0.03 | 0.02 | 0.02, 0.03 |
|  | 5 | 0.03 | 0.02, 0.03 | 0.03 | 0.02, 0.03 | 0.03 | 0.02, 0.03 | 0.03 | 0.02, 0.03 | 0.03 | 0.02, 0.03 | 0.03 | 0.02, 0.03 |
|  | 10 | 0.03 | 0.03, 0.03 | 0.03 | 0.03, 0.03 | 0.03 | 0.03, 0.03 | 0.03 | 0.03, 0.03 | 0.03 | 0.02, 0.03 | 0.03 | 0.02, 0.03 |
|  | 20 | 0.03 | 0.03, 0.03 | 0.03 | 0.03, 0.03 | 0.03 | 0.03, 0.03 | 0.03 | 0.03, 0.03 | 0.03 | 0.02, 0.03 | 0.03 | 0.02, 0.03 |
|  | 40 | 0.03 | 0.03, 0.04 | 0.03 | 0.03, 0.04 | 0.03 | 0.03, 0.04 | 0.03 | 0.03, 0.03 | 0.03 | 0.03, 0.03 | 0.03 | 0.03, 0.03 |
|  | 60 | 0.04 | 0.04, 0.04 | 0.04 | 0.04, 0.04 | 0.04 | 0.04, 0.04 | 0.04 | 0.04, 0.04 | 0.03 | 0.03, 0.04 | 0.03 | 0.03, 0.03 |
|  | 80 | 0.06 | 0.06, 0.06 | 0.06 | 0.06, 0.06 | 0.06 | 0.06, 0.06 | 0.05 | 0.05, 0.06 | 0.04 | 0.04, 0.04 | 0.03 | 0.03, 0.03 |
|  | 90 | 0.08 | 0.08, 0.09 | 0.08 | 0.08, 0.09 | 0.08 | 0.08, 0.08 | 0.08 | 0.07, 0.08 | 0.05 | 0.05, 0.06 | 0.04 | 0.04, 0.04 |
|  |  |  |  |  |  |  |  |  |  |  |  |  |  |
| Relative error | 1 | 2.45 | -2.04, 6.95 | 2.42 | -2.08, 6.91 | 2.65 | -1.86, 7.15 | 2.45 | -2.05, 6.95 | 2.39 | -2.10, 6.89 | 2.33 | -2.16, 6.82 |
|  | 5 | 1.42 | -3.03, 5.88 | 1.39 | -3.06, 5.84 | 1.18 | -3.26, 5.62 | 1.83 | -2.64, 6.30 | 1.96 | -2.52, 6.43 | 1.89 | -2.59, 6.36 |
|  | 10 | 1.20 | -3.24, 5.65 | 1.22 | -3.22, 5.67 | 1.64 | -2.82, 6.11 | 1.43 | -3.02, 5.89 | 1.77 | -2.70, 6.23 | 1.79 | -2.67, 6.26 |
|  | 20 | 3.94 | -0.63, 8.50 | 4.00 | -0.57, 8.56 | 4.30 | -0.28, 8.88 | 5.63 | 0.99, 10.27 | 4.47 | -0.12, 9.05 | 2.64 | -1.87, 7.14 |
|  | 40 | -1.16 | -5.51, 3.18 | -1.17 | -5.51, 3.17 | -1.38 | -5.71, 2.95 | 0.51 | -3.90, 4.93 | -1.05 | -5.39, 3.29 | 0.15 | -4.25, 4.55 |
|  | 60 | 2.40 | -2.10, 6.91 | 2.65 | -1.86, 7.17 | 2.22 | -2.27, 6.72 | 3.91 | -0.66, 8.48 | -0.32 | -4.70, 4.05 | 1.01 | -3.43, 5.44 |
|  | 80 | 2.11 | -2.40, 6.61 | 2.46 | -2.06, 6.98 | 1.73 | -2.76, 6.22 | 4.91 | 0.28, 9.53 | 2.98 | -1.55, 7.52 | 0.94 | -3.50, 5.38 |
|  | 90 | 1.38 | -3.13, 5.88 | 2.73 | -1.83, 7.30 | 2.03 | -2.50, 6.56 | 2.07 | -2.47, 6.60 | 6.09 | 1.39, 10.79 | 4.30 | -0.30, 8.91 |
|  |  |  |  |  |  |  |  |  |  |  |  |  |  |
| Coverage | 1 | 95.10 | 93.76, 96.44 | 95.10 | 93.76, 96.44 | 95.00 | 93.65, 96.35 | 95.00 | 93.65, 96.35 | 95.10 | 93.76, 96.44 | 95.30 | 93.99, 96.61 |
|  | 5 | 91.40 | 89.66, 93.14 | 91.20 | 89.44, 92.96 | 91.50 | 89.77, 93.23 | 95.00 | 93.65, 96.35 | 95.00 | 93.65, 96.35 | 95.60 | 94.33, 96.87 |
|  | 10 | 85.80 | 83.64, 87.96 | 86.00 | 83.85, 88.15 | 86.00 | 83.85, 88.15 | 95.70 | 94.44, 96.96 | 95.10 | 93.76, 96.44 | 95.90 | 94.67, 97.13 |
|  | 20 | 73.30 | 70.56, 76.04 | 72.90 | 70.15, 75.65 | 72.60 | 69.84, 75.36 | 97.00 | 95.94, 98.06 | 96.10 | 94.90, 97.30 | 96.00 | 94.79, 97.21 |
|  | 40 | 61.80 | 58.79, 64.81 | 61.40 | 58.38, 64.42 | 60.90 | 57.88, 63.92 | 94.30 | 92.86, 95.74 | 94.30 | 92.86, 95.74 | 95.20 | 93.88, 96.52 |
|  | 60 | 70.40 | 67.57, 73.23 | 71.00 | 68.19, 73.81 | 67.70 | 64.80, 70.60 | 96.00 | 94.79, 97.21 | 94.30 | 92.86, 95.74 | 95.40 | 94.10, 96.70 |
|  | 80 | 82.10 | 79.72, 84.48 | 82.20 | 79.83, 84.57 | 82.50 | 80.14, 84.86 | 96.60 | 95.48, 97.72 | 94.80 | 93.42, 96.18 | 94.90 | 93.54, 96.26 |
|  | 90 | 90.20 | 88.36, 92.04 | 90.40 | 88.57, 92.23 | 89.40 | 87.49, 91.31 | 96.00 | 94.79, 97.21 | 96.40 | 95.25, 97.55 | 96.10 | 94.90, 97.30 |

CCA – Complete case analysis; FMI- Fraction of Missing Information; SE – Standard error; R^2^ – the squared coefficient of multiple correlation

^a^ For FMI the estimate was the median across simulations and the interval represents the interquartile range. For all other statistics the mean across simulations was taken and the 95% confidence interval was calculated using Monte Carlo standard error.

# Performance statistics for the constant coefficient in the simulation study

Table S4. Performance Statistics for the MCAR Results of the Constant Coefficient in the Simulation Study.

| Statistic | % missing data | CCA | | Model 1  No aux info, R^2^=0.36 | | Model 2  R^2^=0.40 | | Model 3  R^2^=0.52 | | Model 4  R^2^=0.76 | | Model 5  R^2^=0.92 | |
| --- | --- | --- | --- | --- | --- | --- | --- | --- | --- | --- | --- | --- | --- |
| FMI ^a^ | 1 |  |  | 0.01 | 0.01, 0.01 | 0.01 | 0.01, 0.01 | 0.01 | 0.01, 0.01 | 0.00 | 0.00, 0.00 | 0.00 | 0.00, 0.00 |
|  | 5 |  |  | 0.05 | 0.05, 0.05 | 0.05 | 0.05, 0.05 | 0.04 | 0.04, 0.04 | 0.02 | 0.02, 0.02 | 0.01 | 0.01, 0.01 |
|  | 10 |  |  | 0.11 | 0.11, 0.11 | 0.09 | 0.09, 0.09 | 0.07 | 0.07, 0.07 | 0.04 | 0.04, 0.04 | 0.01 | 0.01, 0.02 |
|  | 20 |  |  | 0.20 | 0.20, 0.20 | 0.18 | 0.18, 0.18 | 0.16 | 0.16, 0.16 | 0.08 | 0.08, 0.08 | 0.03 | 0.03, 0.03 |
|  | 40 |  |  | 0.38 | 0.38, 0.38 | 0.35 | 0.35, 0.36 | 0.33 | 0.32, 0.34 | 0.19 | 0.18, 0.20 | 0.08 | 0.07, 0.08 |
|  | 60 |  |  | 0.61 | 0.61, 0.61 | 0.60 | 0.59, 0.60 | 0.53 | 0.52, 0.54 | 0.36 | 0.34, 0.37 | 0.16 | 0.15, 0.17 |
|  | 80 |  |  | 0.80 | 0.80, 0.80 | 0.79 | 0.78, 0.79 | 0.74 | 0.73, 0.75 | 0.61 | 0.59, 0.62 | 0.34 | 0.32, 0.36 |
|  | 90 |  |  | 0.91 | 0.91, 0.91 | 0.90 | 0.90, 0.90 | 0.87 | 0.86, 0.88 | 0.77 | 0.76, 0.79 | 0.56 | 0.53, 0.58 |
|  |  |  |  |  |  |  |  |  |  |  |  |  |  |
| Bias | 1 | 0.00 | 0.00, 0.00 | 0.00 | 0.00, 0.00 | 0.00 | 0.00, 0.00 | 0.00 | 0.00, 0.00 | 0.00 | 0.00, 0.00 | 0.00 | 0.00, 0.00 |
|  | 5 | 0.00 | 0.00, 0.00 | 0.00 | 0.00, 0.00 | 0.00 | 0.00, 0.00 | 0.00 | 0.00, 0.00 | 0.00 | 0.00, 0.00 | 0.00 | 0.00, 0.00 |
|  | 10 | 0.00 | 0.00, 0.00 | 0.00 | 0.00, 0.00 | 0.00 | 0.00, 0.00 | 0.00 | 0.00, 0.00 | 0.00 | 0.00, 0.00 | 0.00 | 0.00, 0.00 |
|  | 20 | 0.00 | 0.00, 0.00 | 0.00 | 0.00, 0.00 | 0.00 | 0.00, 0.00 | 0.00 | 0.00, 0.00 | 0.00 | 0.00, 0.00 | 0.00 | 0.00, 0.00 |
|  | 40 | 0.00 | 0.00, 0.00 | 0.00 | 0.00, 0.00 | 0.00 | 0.00, 0.00 | 0.00 | 0.00, 0.00 | 0.00 | 0.00, 0.00 | 0.00 | 0.00, 0.00 |
|  | 60 | 0.00 | 0.00, 0.00 | 0.00 | 0.00, 0.00 | 0.00 | 0.00, 0.00 | 0.00 | 0.00, 0.00 | 0.00 | 0.00, 0.00 | 0.00 | 0.00, 0.00 |
|  | 80 | 0.00 | -0.01, 0.00 | 0.00 | -0.01, 0.00 | 0.00 | -0.01, 0.00 | 0.00 | -0.01, 0.00 | 0.00 | 0.00, 0.00 | 0.00 | 0.00, 0.00 |
|  | 90 | 0.00 | 0.00, 0.01 | 0.01 | 0.00, 0.01 | 0.00 | 0.00, 0.01 | 0.00 | 0.00, 0.01 | 0.00 | 0.00, 0.00 | 0.00 | 0.00, 0.00 |
|  |  |  |  |  |  |  |  |  |  |  |  |  |  |
| Empirical SE | 1 | 0.03 | 0.02, 0.03 | 0.03 | 0.02, 0.03 | 0.03 | 0.02, 0.03 | 0.03 | 0.02, 0.03 | 0.03 | 0.02, 0.03 | 0.03 | 0.02, 0.03 |
|  | 5 | 0.03 | 0.02, 0.03 | 0.03 | 0.02, 0.03 | 0.03 | 0.02, 0.03 | 0.03 | 0.02, 0.03 | 0.03 | 0.02, 0.03 | 0.03 | 0.02, 0.03 |
|  | 10 | 0.03 | 0.02, 0.03 | 0.03 | 0.02, 0.03 | 0.03 | 0.02, 0.03 | 0.03 | 0.02, 0.03 | 0.03 | 0.02, 0.03 | 0.03 | 0.02, 0.03 |
|  | 20 | 0.03 | 0.03, 0.03 | 0.03 | 0.03, 0.03 | 0.03 | 0.03, 0.03 | 0.03 | 0.03, 0.03 | 0.03 | 0.03, 0.03 | 0.03 | 0.03, 0.03 |
|  | 40 | 0.03 | 0.03, 0.03 | 0.03 | 0.03, 0.03 | 0.03 | 0.03, 0.03 | 0.03 | 0.03, 0.03 | 0.03 | 0.03, 0.03 | 0.03 | 0.03, 0.03 |
|  | 60 | 0.04 | 0.04, 0.04 | 0.04 | 0.04, 0.04 | 0.04 | 0.04, 0.04 | 0.04 | 0.04, 0.04 | 0.03 | 0.03, 0.03 | 0.03 | 0.03, 0.03 |
|  | 80 | 0.06 | 0.05, 0.06 | 0.06 | 0.05, 0.06 | 0.06 | 0.05, 0.06 | 0.05 | 0.05, 0.05 | 0.04 | 0.04, 0.04 | 0.03 | 0.03, 0.03 |
|  | 90 | 0.08 | 0.08, 0.08 | 0.08 | 0.08, 0.08 | 0.08 | 0.07, 0.08 | 0.07 | 0.07, 0.07 | 0.05 | 0.05, 0.06 | 0.04 | 0.04, 0.04 |
|  |  |  |  |  |  |  |  |  |  |  |  |  |  |
| Relative error | 1 | 1.26 | -3.18, 5.70 | 1.23 | -3.21, 5.68 | 1.28 | -3.16, 5.72 | 1.39 | -3.05, 5.84 | 1.23 | -3.21, 5.67 | 1.35 | -3.09, 5.80 |
|  | 5 | 1.67 | -2.79, 6.13 | 1.78 | -2.68, 6.25 | 1.46 | -2.99, 5.91 | 1.69 | -2.77, 6.15 | 1.54 | -2.92, 5.99 | 1.35 | -3.10, 5.80 |
|  | 10 | 2.33 | -2.16, 6.82 | 2.85 | -1.66, 7.36 | 1.86 | -2.61, 6.32 | 2.07 | -2.41, 6.54 | 2.26 | -2.23, 6.75 | 1.56 | -2.89, 6.02 |
|  | 20 | -1.02 | -5.37, 3.32 | -1.21 | -5.55, 3.12 | -1.78 | -6.09, 2.53 | 0.02 | -4.36, 4.41 | -1.03 | -5.37, 3.31 | -2.82 | -7.09, 1.44 |
|  | 40 | -1.11 | -5.45, 3.23 | -2.51 | -6.79, 1.77 | -3.90 | -8.12, 0.32 | -0.94 | -5.29, 3.40 | -1.74 | -6.05, 2.57 | -3.09 | -7.34, 1.16 |
|  | 60 | 0.23 | -4.17, 4.63 | 2.02 | -2.46, 6.50 | 1.78 | -2.68, 6.25 | -1.77 | -6.08, 2.55 | -0.74 | -5.09, 3.62 | -1.90 | -6.21, 2.40 |
|  | 80 | 1.03 | -3.41, 5.47 | 0.14 | -4.26, 4.55 | -0.10 | -4.49, 4.29 | -1.74 | -6.06, 2.58 | 0.43 | -3.98, 4.85 | -0.11 | -4.49, 4.28 |
|  | 90 | 1.52 | -2.96, 5.99 | 5.43 | 0.79, 10.08 | 4.61 | 0.00, 9.22 | 0.16 | -4.25, 4.57 | 0.55 | -3.88, 4.97 | 2.73 | -1.78, 7.25 |
|  |  |  |  |  |  |  |  |  |  |  |  |  |  |
| Coverage | 1 | 96.00 | 94.79, 97.21 | 96.00 | 94.79, 97.21 | 95.80 | 94.56, 97.04 | 95.80 | 94.56, 97.04 | 95.90 | 94.67, 97.13 | 95.40 | 94.10, 96.70 |
|  | 5 | 96.30 | 95.13, 97.47 | 96.40 | 95.25, 97.55 | 96.40 | 95.25, 97.55 | 96.20 | 95.01, 97.39 | 95.70 | 94.44, 96.96 | 95.80 | 94.56, 97.04 |
|  | 10 | 95.70 | 94.44, 96.96 | 95.80 | 94.56, 97.04 | 95.20 | 93.88, 96.52 | 95.60 | 94.33, 96.87 | 95.60 | 94.33, 96.87 | 95.70 | 94.44, 96.96 |
|  | 20 | 94.00 | 92.53, 95.47 | 93.90 | 92.42, 95.38 | 93.50 | 91.97, 95.03 | 94.60 | 93.20, 96.00 | 94.50 | 93.09, 95.91 | 93.80 | 92.31, 95.29 |
|  | 40 | 94.50 | 93.09, 95.91 | 94.00 | 92.53, 95.47 | 93.70 | 92.19, 95.21 | 94.30 | 92.86, 95.74 | 93.80 | 92.31, 95.29 | 93.70 | 92.19, 95.21 |
|  | 60 | 95.20 | 93.88, 96.52 | 95.50 | 94.22, 96.78 | 94.90 | 93.54, 96.26 | 94.70 | 93.31, 96.09 | 95.00 | 93.65, 96.35 | 93.80 | 92.31, 95.29 |
|  | 80 | 95.00 | 93.65, 96.35 | 95.00 | 93.65, 96.35 | 94.40 | 92.97, 95.83 | 94.20 | 92.75, 95.65 | 94.60 | 93.20, 96.00 | 94.50 | 93.09, 95.91 |
|  | 90 | 95.00 | 93.65, 96.35 | 95.60 | 94.33, 96.87 | 95.00 | 93.65, 96.35 | 93.80 | 92.31, 95.29 | 94.90 | 93.54, 96.26 | 95.90 | 94.67, 97.13 |

CCA – Complete case analysis; FMI- Fraction of Missing Information; SE – Standard error; R^2^ – the squared coefficient of multiple correlation

^a^ For FMI the estimate was the median across simulations and the interval represents the interquartile range. For all other statistics the mean across simulations was taken and the 95% confidence interval was calculated using Monte Carlo standard error.

Table S5. Performance Statistics for the MAR Results of the Constant Coefficient in the Simulation Study.

| Statistic | % missing data | CCA | | Model 1  No aux info, R^2^=0.36 | | Model 2  R^2^=0.40 | | Model 3  R^2^=0.52 | | Model 4  R^2^=0.76 | | Model 5  R^2^=0.92 | |
| --- | --- | --- | --- | --- | --- | --- | --- | --- | --- | --- | --- | --- | --- |
| FMI ^a^ | 1 | 0.01 | 0.01, 0.01 | 0.01 | 0.01, 0.01 | 0.01 | 0.01, 0.01 | 0.00 | 0.00, 0.00 | 0.00 | 0.00, 0.00 | 0.01 | 0.01, 0.01 |
|  | 5 | 0.05 | 0.05, 0.06 | 0.05 | 0.04, 0.05 | 0.04 | 0.04, 0.04 | 0.02 | 0.02, 0.02 | 0.01 | 0.01, 0.01 | 0.05 | 0.05, 0.06 |
|  | 10 | 0.11 | 0.10, 0.12 | 0.11 | 0.10, 0.11 | 0.09 | 0.09, 0.10 | 0.05 | 0.04, 0.05 | 0.02 | 0.01, 0.02 | 0.11 | 0.10, 0.12 |
|  | 20 | 0.22 | 0.21, 0.23 | 0.21 | 0.20, 0.22 | 0.19 | 0.18, 0.21 | 0.11 | 0.10, 0.12 | 0.04 | 0.04, 0.04 | 0.22 | 0.21, 0.23 |
|  | 40 | 0.45 | 0.43, 0.46 | 0.43 | 0.42, 0.45 | 0.44 | 0.42, 0.46 | 0.28 | 0.27, 0.30 | 0.12 | 0.11, 0.13 | 0.45 | 0.43, 0.46 |
|  | 60 | 0.68 | 0.66, 0.69 | 0.66 | 0.64, 0.68 | 0.68 | 0.67, 0.70 | 0.52 | 0.50, 0.54 | 0.26 | 0.25, 0.28 | 0.68 | 0.66, 0.69 |
|  | 80 | 0.86 | 0.85, 0.87 | 0.86 | 0.84, 0.87 | 0.88 | 0.87, 0.89 | 0.78 | 0.76, 0.80 | 0.55 | 0.52, 0.58 | 0.86 | 0.85, 0.87 |
|  | 90 | 0.94 | 0.93, 0.95 | 0.94 | 0.93, 0.94 | 0.95 | 0.94, 0.96 | 0.90 | 0.89, 0.91 | 0.77 | 0.73, 0.79 | 0.94 | 0.93, 0.95 |
|  |  |  |  |  |  |  |  |  |  |  |  |  |  |
| Bias | 1 | 0.00 | 0.00, 0.00 | 0.00 | 0.00, 0.00 | 0.00 | 0.00, 0.00 | 0.00 | 0.00, 0.00 | 0.00 | 0.00, 0.00 | 0.00 | 0.00, 0.00 |
|  | 5 | 0.02 | 0.02, 0.02 | 0.02 | 0.02, 0.02 | 0.02 | 0.02, 0.02 | 0.00 | 0.00, 0.00 | 0.00 | 0.00, 0.00 | 0.00 | 0.00, 0.00 |
|  | 10 | 0.03 | 0.03, 0.04 | 0.03 | 0.03, 0.04 | 0.03 | 0.03, 0.04 | 0.00 | 0.00, 0.00 | 0.00 | 0.00, 0.00 | 0.00 | 0.00, 0.00 |
|  | 20 | 0.07 | 0.07, 0.07 | 0.07 | 0.07, 0.07 | 0.07 | 0.07, 0.07 | 0.00 | 0.00, 0.00 | 0.00 | 0.00, 0.00 | 0.00 | 0.00, 0.00 |
|  | 40 | 0.13 | 0.13, 0.13 | 0.13 | 0.13, 0.13 | 0.13 | 0.13, 0.13 | 0.00 | 0.00, 0.00 | 0.00 | 0.00, 0.00 | 0.00 | 0.00, 0.00 |
|  | 60 | 0.20 | 0.20, 0.20 | 0.20 | 0.20, 0.20 | 0.20 | 0.20, 0.20 | 0.00 | 0.00, 0.00 | 0.00 | 0.00, 0.00 | 0.00 | 0.00, 0.00 |
|  | 80 | 0.28 | 0.28, 0.29 | 0.28 | 0.28, 0.29 | 0.28 | 0.28, 0.29 | 0.00 | 0.00, 0.00 | 0.00 | -0.01, 0.00 | 0.00 | 0.00, 0.00 |
|  | 90 | 0.34 | 0.33, 0.34 | 0.33 | 0.33, 0.34 | 0.33 | 0.33, 0.34 | 0.00 | 0.00, 0.01 | 0.00 | -0.01, 0.00 | 0.00 | -0.01, 0.00 |
|  |  |  |  |  |  |  |  |  |  |  |  |  |  |
| Empirical SE | 1 | 0.03 | 0.02, 0.03 | 0.03 | 0.02, 0.03 | 0.03 | 0.02, 0.03 | 0.03 | 0.02, 0.03 | 0.03 | 0.02, 0.03 | 0.03 | 0.02, 0.03 |
|  | 5 | 0.03 | 0.03, 0.03 | 0.03 | 0.03, 0.03 | 0.03 | 0.03, 0.03 | 0.03 | 0.03, 0.03 | 0.03 | 0.02, 0.03 | 0.03 | 0.02, 0.03 |
|  | 10 | 0.03 | 0.03, 0.03 | 0.03 | 0.03, 0.03 | 0.03 | 0.03, 0.03 | 0.03 | 0.03, 0.03 | 0.03 | 0.03, 0.03 | 0.03 | 0.03, 0.03 |
|  | 20 | 0.03 | 0.03, 0.03 | 0.03 | 0.03, 0.03 | 0.03 | 0.03, 0.03 | 0.03 | 0.03, 0.03 | 0.03 | 0.03, 0.03 | 0.03 | 0.03, 0.03 |
|  | 40 | 0.03 | 0.03, 0.04 | 0.03 | 0.03, 0.04 | 0.03 | 0.03, 0.04 | 0.03 | 0.03, 0.04 | 0.03 | 0.03, 0.03 | 0.03 | 0.03, 0.03 |
|  | 60 | 0.05 | 0.04, 0.05 | 0.05 | 0.04, 0.05 | 0.04 | 0.04, 0.05 | 0.05 | 0.04, 0.05 | 0.04 | 0.04, 0.04 | 0.03 | 0.03, 0.03 |
|  | 80 | 0.07 | 0.06, 0.07 | 0.07 | 0.06, 0.07 | 0.06 | 0.06, 0.07 | 0.07 | 0.07, 0.07 | 0.05 | 0.05, 0.06 | 0.04 | 0.04, 0.04 |
|  | 90 | 0.10 | 0.09, 0.10 | 0.10 | 0.09, 0.10 | 0.09 | 0.09, 0.10 | 0.11 | 0.10, 0.11 | 0.08 | 0.08, 0.08 | 0.05 | 0.05, 0.05 |
|  |  |  |  |  |  |  |  |  |  |  |  |  |  |
| Relative error | 1 | -2.35 | -6.64, 1.93 | -2.38 | -6.66, 1.90 | -2.54 | -6.81, 1.74 | -2.21 | -6.50, 2.08 | -2.34 | -6.62, 1.95 | -2.51 | -6.78, 1.77 |
|  | 5 | -1.19 | -5.53, 3.14 | -1.18 | -5.51, 3.16 | -1.36 | -5.69, 2.96 | -1.60 | -5.92, 2.72 | -1.80 | -6.11, 2.51 | -2.29 | -6.58, 1.99 |
|  | 10 | -2.90 | -7.16, 1.36 | -2.84 | -7.10, 1.42 | -2.21 | -6.50, 2.08 | -2.73 | -6.99, 1.54 | -2.78 | -7.04, 1.49 | -2.66 | -6.93, 1.61 |
|  | 20 | -3.16 | -7.41, 1.09 | -3.03 | -7.29, 1.22 | -3.21 | -7.46, 1.03 | -3.84 | -8.06, 0.38 | -2.86 | -7.12, 1.41 | -3.30 | -7.54, 0.95 |
|  | 40 | -1.98 | -6.28, 2.32 | -2.49 | -6.77, 1.79 | -2.76 | -7.03, 1.51 | -1.89 | -6.20, 2.42 | -1.41 | -5.74, 2.91 | -2.71 | -6.97, 1.56 |
|  | 60 | -3.96 | -8.18, 0.26 | -3.84 | -8.07, 0.39 | -3.10 | -7.36, 1.16 | -1.48 | -5.81, 2.85 | -3.53 | -7.77, 0.71 | -0.31 | -4.68, 4.07 |
|  | 80 | 1.32 | -3.14, 5.79 | 1.87 | -2.62, 6.36 | 1.78 | -2.70, 6.27 | 3.88 | -0.70, 8.46 | 2.58 | -1.94, 7.10 | 1.41 | -3.05, 5.87 |
|  | 90 | 3.09 | -1.48, 7.67 | 4.35 | -0.28, 8.98 | 3.72 | -0.88, 8.32 | 2.45 | -2.10, 7.00 | 1.57 | -2.94, 6.07 | 1.58 | -2.92, 6.08 |
|  |  |  |  |  |  |  |  |  |  |  |  |  |  |
| Coverage | 1 | 93.80 | 92.31, 95.29 | 93.80 | 92.31, 95.29 | 93.70 | 92.19, 95.21 | 94.20 | 92.75, 95.65 | 94.30 | 92.86, 95.74 | 94.00 | 92.53, 95.47 |
|  | 5 | 89.20 | 87.28, 91.12 | 89.20 | 87.28, 91.12 | 89.10 | 87.17, 91.03 | 94.10 | 92.64, 95.56 | 94.40 | 92.97, 95.83 | 94.30 | 92.86, 95.74 |
|  | 10 | 74.40 | 71.70, 77.10 | 74.60 | 71.90, 77.30 | 74.50 | 71.80, 77.20 | 93.80 | 92.31, 95.29 | 93.70 | 92.19, 95.21 | 93.80 | 92.31, 95.29 |
|  | 20 | 33.70 | 30.77, 36.63 | 33.80 | 30.87, 36.73 | 31.40 | 28.52, 34.28 | 94.30 | 92.86, 95.74 | 94.30 | 92.86, 95.74 | 94.00 | 92.53, 95.47 |
|  | 40 | 3.20 | 2.11, 4.29 | 2.90 | 1.86, 3.94 | 2.40 | 1.45, 3.35 | 94.10 | 92.64, 95.56 | 95.20 | 93.88, 96.52 | 94.60 | 93.20, 96.00 |
|  | 60 | 0.70 | 0.18, 1.22 | 0.70 | 0.18, 1.22 | 1.00 | 0.38, 1.62 | 95.00 | 93.65, 96.35 | 94.30 | 92.86, 95.74 | 94.70 | 93.31, 96.09 |
|  | 80 | 1.50 | 0.75, 2.25 | 1.50 | 0.75, 2.25 | 1.10 | 0.45, 1.75 | 95.90 | 94.67, 97.13 | 95.50 | 94.22, 96.78 | 95.80 | 94.56, 97.04 |
|  | 90 | 9.10 | 7.32, 10.88 | 9.10 | 7.32, 10.88 | 8.00 | 6.32, 9.68 | 95.90 | 94.67, 97.13 | 94.80 | 93.42, 96.18 | 95.70 | 94.44, 96.96 |

CCA – Complete case analysis; FMI- Fraction of Missing Information; SE – Standard error; R^2^ – the squared coefficient of multiple correlation

^a^ For FMI the estimate was the median across simulations and the interval represents the interquartile range. For all other statistics the mean across simulations was taken and the 95% confidence interval was calculated using Monte Carlo standard error.

# FMI and efficiency gains were not sensitive to whether the auxiliary variable was included in the missingness mechanism

In imputation model 3 the auxiliary variable $Z_{1}$ can be replaced by $Z_{2}$ and still provide the same $R_{Y}^{2}$ value. However, the variable $Z_{1}$ is associated with the missing data mechanism while $Z_{2}$ is not. These two models can be used to check whether parameters are sensitive to the auxiliary variable being associated with the missing data mechanism. Table S6 shows that FMI and empirical SE do not differ between the two models. However, because Z1 is associated with missingness, model 3 (using $Z_{2}$ in place of $Z_{1}$) is biased where the original model 3 was not.

Table S6. Performance Statistics for the MAR Results of the Exposure Coefficient of Model 3 replacing variable Z_1_ with Z_2_.

| Statistic | % missing data | CCA | | Model 3  R^2^=0.52 | | Model 3 using Z2  R^2^=0.52 | |
| --- | --- | --- | --- | --- | --- | --- | --- |
| FMI ^a^ | 1 |  |  | 0.01 | 0.01, 0.02 | 0.01 | 0.01, 0.02 |
|  | 5 |  |  | 0.06 | 0.05, 0.07 | 0.06 | 0.05, 0.07 |
|  | 10 |  |  | 0.12 | 0.11, 0.14 | 0.12 | 0.11, 0.13 |
|  | 20 |  |  | 0.22 | 0.21, 0.24 | 0.22 | 0.20, 0.23 |
|  | 40 |  |  | 0.42 | 0.40, 0.44 | 0.40 | 0.38, 0.42 |
|  | 60 |  |  | 0.60 | 0.58, 0.62 | 0.59 | 0.57, 0.61 |
|  | 80 |  |  | 0.79 | 0.77, 0.81 | 0.78 | 0.76, 0.80 |
|  | 90 |  |  | 0.89 | 0.87, 0.90 | 0.89 | 0.87, 0.90 |
|  |  |  |  |  |  |  |  |
| Bias | 1 | 0.00 | -0.01, 0.00 | 0.00 | 0.00, 0.00 | 0.00 | -0.01, 0.00 |
|  | 5 | -0.01 | -0.02, -0.01 | 0.00 | 0.00, 0.00 | -0.01 | -0.02, -0.01 |
|  | 10 | -0.02 | -0.03, -0.02 | 0.00 | 0.00, 0.00 | -0.02 | -0.03, -0.02 |
|  | 20 | -0.04 | -0.04, -0.04 | 0.00 | 0.00, 0.00 | -0.04 | -0.04, -0.04 |
|  | 40 | -0.06 | -0.06, -0.06 | 0.00 | 0.00, 0.00 | -0.06 | -0.06, -0.05 |
|  | 60 | -0.06 | -0.07, -0.06 | 0.00 | 0.00, 0.00 | -0.06 | -0.07, -0.06 |
|  | 80 | -0.06 | -0.07, -0.06 | 0.00 | -0.01, 0.00 | -0.06 | -0.07, -0.06 |
|  | 90 | -0.05 | -0.06, -0.05 | 0.00 | 0.00, 0.00 | -0.05 | -0.05, -0.05 |
|  |  |  |  |  |  |  |  |
| Empirical SE | 1 | 0.02 | 0.02, 0.03 | 0.02 | 0.02, 0.03 | 0.02 | 0.02, 0.03 |
|  | 5 | 0.03 | 0.02, 0.03 | 0.03 | 0.02, 0.03 | 0.03 | 0.02, 0.03 |
|  | 10 | 0.03 | 0.03, 0.03 | 0.03 | 0.03, 0.03 | 0.03 | 0.03, 0.03 |
|  | 20 | 0.03 | 0.03, 0.03 | 0.03 | 0.03, 0.03 | 0.03 | 0.03, 0.03 |
|  | 40 | 0.03 | 0.03, 0.04 | 0.03 | 0.03, 0.03 | 0.03 | 0.03, 0.03 |
|  | 60 | 0.04 | 0.04, 0.04 | 0.04 | 0.04, 0.04 | 0.04 | 0.04, 0.04 |
|  | 80 | 0.06 | 0.06, 0.06 | 0.05 | 0.05, 0.06 | 0.05 | 0.05, 0.06 |
|  | 90 | 0.08 | 0.08, 0.09 | 0.08 | 0.07, 0.08 | 0.07 | 0.07, 0.07 |
|  |  |  |  |  |  |  |  |
| Relative error | 1 | 2.45 | -2.04, 6.95 | 2.45 | -2.05, 6.95 | 2.15 | -2.33, 6.64 |
|  | 5 | 1.42 | -3.03, 5.88 | 1.83 | -2.64, 6.30 | 2.22 | -2.26, 6.71 |
|  | 10 | 1.20 | -3.24, 5.65 | 1.43 | -3.02, 5.89 | 1.60 | -2.86, 6.06 |
|  | 20 | 3.94 | -0.63, 8.50 | 5.63 | 0.99, 10.27 | 3.06 | -1.46, 7.59 |
|  | 40 | -1.16 | -5.51, 3.18 | 0.51 | -3.90, 4.93 | -1.42 | -5.75, 2.91 |
|  | 60 | 2.40 | -2.10, 6.91 | 3.91 | -0.66, 8.48 | 1.09 | -3.36, 5.53 |
|  | 80 | 2.11 | -2.40, 6.61 | 4.91 | 0.28, 9.53 | 1.32 | -3.15, 5.79 |
|  | 90 | 1.38 | -3.13, 5.88 | 2.07 | -2.47, 6.60 | 5.84 | 1.14, 10.54 |
|  |  |  |  |  |  |  |  |
| Coverage | 1 | 95.10 | 93.76, 96.44 | 95.00 | 93.65, 96.35 | 95.3 | 93.99, 96.61 |
|  | 5 | 91.40 | 89.66, 93.14 | 95.00 | 93.65, 96.35 | 91.6 | 89.88, 93.32 |
|  | 10 | 85.80 | 83.64, 87.96 | 95.70 | 94.44, 96.96 | 84.8 | 82.57, 87.03 |
|  | 20 | 73.30 | 70.56, 76.04 | 97.00 | 95.94, 98.06 | 71.6 | 68.81, 74.39 |
|  | 40 | 61.80 | 58.79, 64.81 | 94.30 | 92.86, 95.74 | 59.5 | 56.46, 62.54 |
|  | 60 | 70.40 | 67.57, 73.23 | 96.00 | 94.79, 97.21 | 64.6 | 61.64, 67.56 |
|  | 80 | 82.10 | 79.72, 84.48 | 96.60 | 95.48, 97.72 | 80.1 | 77.63, 82.57 |
|  | 90 | 90.20 | 88.36, 92.04 | 96.00 | 94.79, 97.21 | 90.1 | 88.25, 91.95 |

CCA – Complete case analysis; FMI- Fraction of Missing Information; SE – Standard error; R^2^ – the squared coefficient of multiple correlation

^a^ For FMI the estimate was the median across simulations and the interval represents the interquartile range. For all other statistics the mean across simulations was taken and the 95% confidence interval was calculated using Monte Carlo standard error.

# Missing data in included and excluded sample for the applied example

Table S7. Quantity of Missing Variables (Outcome, Exposure or Auxiliary) Separated by Inclusion in the Sample.

|  | N(%) | | |
| --- | --- | --- | --- |
| Number of missing variables (outcome, exposure or auxiliary) | Missing confounders/  excluded | No missing confounders/  included | Total |
| 0 - No missing | 42 (1.51) | 1,607 (13.49) | 1,649 (11.22) |
| 1 | 142 (5.11) | 1,408 (11.82) | 1,550 (10.55) |
| 2 | 201 (7.23) | 1,292 (10.85) | 1,493 (10.16) |
| 3 | 461 (16.58) | 1,817 (15.25) | 2,278 (15.51) |
| 4 | 482 (17.34) | 1,771 (14.87) | 2,253 (15.34) |
| 5 | 249 (8.96) | 1,174 (9.86) | 1,423 (9.69) |
| 6 | 257 (9.24) | 1,118 (9.39) | 1,375 (9.36) |
| 7 | 551 (19.82) | 1,724 (14.47) | 2,275 (15.49) |
| 8 - All missing | 395 (14.21) | 0 (0.00) | 395 (2.69) |
| Total | 2,780 (100.00) | 11,911 (100.00) | 14,691 (100.00) |

# Missing data pattern in the applied example

Table S8. Missing Data Pattern for the Exposure, Outcome and the Strongest Auxiliary for the Outcome

| Pattern | Exposure | Outcome | Strongest auxiliary ^a^ | N(%) |
| --- | --- | --- | --- | --- |
| 1 | Complete | Missing | Missing | 4,803 (40.32) |
| 2 | Complete | Complete | Complete | 3,974 (33.36) |
| 3 | Complete | Missing | Complete | 2,698 (22.65) |
| 4 | Complete | Complete | Missing | 496 (4.16) |

^a^ The strongest auxiliary for predicting the outcome was IQ at age 8

# Applied example exposure coefficient results for the unadjusted model


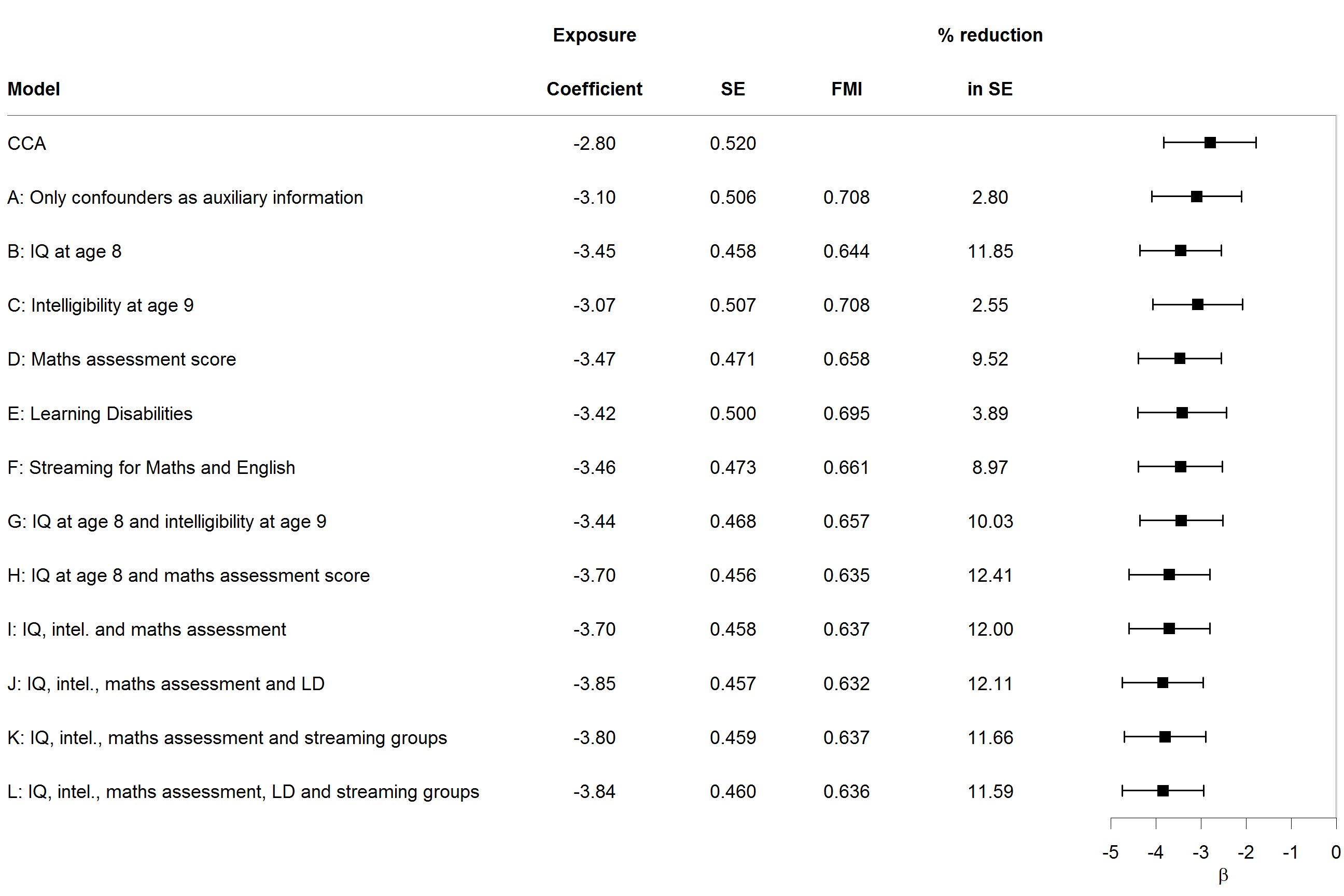


Figure S3. Estimate, standard error and FMI for the exposure coefficient in the applied example unadjusted analysis model. Reduction in SE is relative to CCA. CCA=complete case analysis; FMI= fraction of missing information; SE = standard error.

# Applied example constant coefficient results

Table S9. Results for the Estimate, Standard Error and FMI for the Constant Coefficient in the Unadjusted Analysis Model.

| Model | Estimate | SE | FMI |
| --- | --- | --- | --- |
| CCA | 92.673 | 0.2116 |  |
| A | 91.665 | 0.2123 | 0.585 |
| B | 91.205 | 0.1971 | 0.517 |
| C | 91.607 | 0.2081 | 0.566 |
| D | 91.219 | 0.2025 | 0.537 |
| E | 91.507 | 0.2152 | 0.587 |
| F | 91.355 | 0.2072 | 0.557 |
| G | 91.167 | 0.1987 | 0.524 |
| H | 90.925 | 0.1993 | 0.521 |
| I | 90.893 | 0.1985 | 0.517 |
| J | 90.843 | 0.1975 | 0.506 |
| K | 90.807 | 0.1953 | 0.496 |
| L | 90.801 | 0.1953 | 0.494 |

CCA = complete case analysis; FMI= fraction of missing information; SE = standard error.

Table S10. Results for the Estimate, Standard Error and FMI for the Constant Coefficient in the Adjusted Analysis Model.

| Model | Estimate | SE | FMI |
| --- | --- | --- | --- |
| CCA | 84.264 | 0.8162 |  |
| A | 84.246 | 0.8250 | 0.716 |
| B | 84.020 | 0.7752 | 0.678 |
| C | 84.121 | 0.8256 | 0.715 |
| D | 83.872 | 0.7938 | 0.690 |
| E | 84.308 | 0.8253 | 0.711 |
| F | 84.369 | 0.8195 | 0.708 |
| G | 83.984 | 0.7706 | 0.673 |
| H | 83.693 | 0.7295 | 0.633 |
| I | 83.726 | 0.7543 | 0.656 |
| J | 83.676 | 0.7528 | 0.651 |
| K | 83.691 | 0.7596 | 0.659 |
| L | 83.712 | 0.7328 | 0.632 |

CCA = complete case analysis; FMI= fraction of missing information; SE = standard error.

# Simulation study using a binary outcome

Methods

*Data model.* We first simulated 1000 observations of exposure $X$ and auxiliary variables $Z_{1}-Z_{3}$ from independent standard normal distributions. $Y$ was then simulated as a binary outcome variable using a logistic regression model as the linear combination of $X$ and $Z_{1}-Z_{3}$, each with a coefficient of log(2), and a constant of negative log(0.9455/0.0545).

Missingness was simulated in the same way as for our linear example. MCAR data was simulated by removing the first $p$ observations such that $\frac{p}{n}$ gives the required proportion of missing data. MAR missingness was simulated under a logistic regression model using

$\mathrm{logit}\left( \lambda_{i} \right)=\alpha+Z_{1i}+X_{i}$,

where $\alpha$ was manipulated for the different simulation settings to provide the required proportion of missing data on average across datasets.

*Analysis model.* For each simulation setting and imputation model the following logistic regression analysis model was used:

$$\mathrm{logit}\left( E\left[ Y \right] \right)=\beta_{0}+\beta_{1}x,$$

where $\beta_{0}$ and $\beta_{1}$ are the intercept and exposure coefficient respectively. We took the true value of $\beta_{1}$ to be the mean across all simulations of the coefficient obtained from analysis of the full dataset with no observations removed. We used this as the definition of the true value as opposed to the designed value, as was used in the linear model, in order to account for non-collapsibility of the OR for our exposure coefficient.

*Imputation models.* Five imputation models were considered for both MCAR and MAR data (see Table S11). All models contained the variables included in the analysis model and used logistic regression to impute the missing outcome. Model 1 contained no auxiliary information. Model 2 and model 3 were identical in terms of quantity of auxiliary information but Model 3 contained all variables in the missingness mechanism while Model 2 did not. Models 4 and 5 contained increasing quantities of auxiliary information, achieved by increasing the number of $Z$ variables included in the imputation model. To measure the quantity of auxiliary information we have used the sum of the designed odds ratios for $Y$, $\sum OR_{Y}$. The $\sum OR_{Y}$ is used in place of $R_{Y}^{2}$ from the simulation study with a continuous outcome. For each imputation model 1000 imputations were run.

Table S11. Description of the Imputation Models Used for Both MCAR and MAR Data.

| Imputation model | Variables included | $\sum OR_{Y}$^a^ |
| --- | --- | --- |
| 1 (least auxiliary information) | *Y, X* | 2 |
| 2 | *Y, X, Z_2_* | 4 |
| 3 | *Y, X, Z_1_* | 4 |
| 4 | *Y, X, Z_1-2_* | 6 |
| 5 (most auxiliary information) | *Y, X, Z_1-3_* | 8 |

^a^ $\sum OR_{Y}$ is the sum of the designed odds ratios in the logistic model used to produce Y for variables included in the imputation model.

*Comparisons.* We repeated the simulation study for 1%, 5%, 10%, 20%, 40% 60%, 80% and 90% missing data. For all scenarios, we generated 1000 independent simulated datasets We used the same performance statistics for the logistic model as we did for the linear model. All performance statistics were calculated on the log(OR) scale. Performance statistics should not be compared between our logistic and linear simulation studies due to differences between the imputation models used.

Results

We present the results of the simulation study with a binary outcome in Table S12 for MCAR data and Table S13 for MAR data. In the MAR setting bias is reduced, compared to CCA, when all variables in the missingness mechanism are included in the imputation model. However, the quantity of bias is smaller than the size of the empirical SE. Bias can therefore be considered to be negligible in all of our simulation settings. Small improvements in efficiency over CCA were observed with increasing auxiliary information for both MCAR and MAR data.

We display a plot of the empirical SE against the FMI for MCAR data in Figure S4 and MAR data in Figure S5. As in our linear model example, the FMI was reduced with increasing auxiliary information. This reduction was greater for MAR data than for MCAR data.

Table S12. Performance Statistics for the MCAR Results of the Exposure Coefficient in the Binary Outcome Simulation Study.

| Statistic | % missing data | CCA | | Model 1  No aux info, R^2^=0.36 | | Model 2  R^2^=0.40 | | Model 3  R^2^=0.52 | | Model 4  R^2^=0.76 | | Model 5  R^2^=0.92 | |
| --- | --- | --- | --- | --- | --- | --- | --- | --- | --- | --- | --- | --- | --- |
| FMI ^a^ | 1 |  |  | 0.01 | 0.01, 0.01 | 0.01 | 0.01, 0.01 | 0.01 | 0.01, 0.01 | 0.01 | 0.01, 0.01 | 0.01 | 0.00, 0.01 |
|  | 5 |  |  | 0.05 | 0.04, 0.06 | 0.05 | 0.04, 0.06 | 0.05 | 0.04, 0.05 | 0.04 | 0.04, 0.05 | 0.04 | 0.03, 0.05 |
|  | 10 |  |  | 0.10 | 0.09, 0.11 | 0.09 | 0.08, 0.10 | 0.09 | 0.08, 0.10 | 0.09 | 0.08, 0.10 | 0.08 | 0.07, 0.10 |
|  | 20 |  |  | 0.20 | 0.19, 0.22 | 0.19 | 0.18, 0.21 | 0.19 | 0.18, 0.21 | 0.18 | 0.17, 0.20 | 0.17 | 0.16, 0.19 |
|  | 40 |  |  | 0.40 | 0.38, 0.42 | 0.39 | 0.37, 0.41 | 0.38 | 0.37, 0.41 | 0.38 | 0.36, 0.40 | 0.36 | 0.34, 0.38 |
|  | 60 |  |  | 0.60 | 0.58, 0.61 | 0.59 | 0.57, 0.61 | 0.59 | 0.57, 0.61 | 0.58 | 0.56, 0.60 | 0.56 | 0.54, 0.59 |
|  | 80 |  |  | 0.79 | 0.78, 0.81 | 0.78 | 0.77, 0.80 | 0.79 | 0.77, 0.80 | 0.79 | 0.77, 0.81 | 0.77 | 0.75, 0.79 |
|  | 90 |  |  | 0.89 | 0.88, 0.90 | 0.90 | 0.88, 0.91 | 0.89 | 0.88, 0.91 | 0.89 | 0.87, 0.90 | 0.89 | 0.87, 0.90 |
|  |  |  |  |  |  |  |  |  |  |  |  |  |  |
| Bias | 1 | 0.00 | -0.01, 0.01 | 0.00 | -0.01, 0.01 | 0.00 | -0.01, 0.01 | 0.00 | -0.01, 0.01 | 0.00 | -0.01, 0.01 | 0.00 | -0.01, 0.01 |
|  | 5 | 0.00 | -0.01, 0.01 | 0.00 | -0.01, 0.01 | 0.00 | 0.00, 0.01 | 0.00 | -0.01, 0.01 | 0.00 | -0.01, 0.01 | 0.00 | -0.01, 0.01 |
|  | 10 | 0.00 | 0.00, 0.01 | 0.00 | 0.00, 0.01 | 0.00 | 0.00, 0.01 | 0.00 | 0.00, 0.01 | 0.00 | 0.00, 0.01 | 0.00 | 0.00, 0.01 |
|  | 20 | 0.00 | 0.00, 0.01 | 0.00 | -0.01, 0.01 | 0.00 | -0.01, 0.01 | 0.00 | 0.00, 0.01 | 0.00 | -0.01, 0.01 | 0.00 | 0.00, 0.01 |
|  | 40 | 0.00 | -0.01, 0.01 | 0.00 | -0.01, 0.01 | 0.00 | -0.01, 0.01 | 0.00 | -0.01, 0.01 | 0.01 | 0.00, 0.02 | 0.01 | 0.00, 0.02 |
|  | 60 | 0.00 | -0.01, 0.02 | 0.00 | -0.01, 0.02 | 0.01 | 0.00, 0.02 | 0.00 | -0.01, 0.01 | 0.00 | -0.01, 0.01 | 0.00 | -0.01, 0.02 |
|  | 80 | 0.02 | 0.00, 0.04 | 0.02 | 0.01, 0.04 | 0.03 | 0.01, 0.04 | 0.01 | -0.01, 0.02 | 0.01 | -0.01, 0.02 | 0.00 | -0.01, 0.02 |
|  | 90 | 0.04 | 0.02, 0.07 | 0.07 | 0.04, 0.09 | 0.03 | 0.00, 0.05 | 0.05 | 0.02, 0.07 | 0.02 | 0.00, 0.04 | 0.02 | 0.00, 0.04 |
|  |  |  |  |  |  |  |  |  |  |  |  |  |  |
| Empirical SE | 1 | 0.11 | 0.11, 0.12 | 0.11 | 0.11, 0.12 | 0.11 | 0.11, 0.12 | 0.11 | 0.11, 0.12 | 0.11 | 0.11, 0.12 | 0.11 | 0.11, 0.12 |
|  | 5 | 0.11 | 0.11, 0.12 | 0.11 | 0.11, 0.12 | 0.11 | 0.11, 0.12 | 0.11 | 0.11, 0.12 | 0.11 | 0.11, 0.12 | 0.11 | 0.11, 0.12 |
|  | 10 | 0.12 | 0.11, 0.12 | 0.12 | 0.11, 0.12 | 0.12 | 0.11, 0.12 | 0.12 | 0.11, 0.12 | 0.12 | 0.11, 0.12 | 0.12 | 0.11, 0.12 |
|  | 20 | 0.13 | 0.12, 0.13 | 0.13 | 0.12, 0.13 | 0.13 | 0.12, 0.13 | 0.13 | 0.12, 0.13 | 0.12 | 0.12, 0.13 | 0.12 | 0.12, 0.13 |
|  | 40 | 0.14 | 0.14, 0.15 | 0.14 | 0.14, 0.15 | 0.14 | 0.14, 0.15 | 0.14 | 0.14, 0.15 | 0.14 | 0.14, 0.15 | 0.14 | 0.13, 0.15 |
|  | 60 | 0.18 | 0.17, 0.19 | 0.18 | 0.17, 0.19 | 0.17 | 0.17, 0.18 | 0.18 | 0.17, 0.18 | 0.17 | 0.17, 0.18 | 0.17 | 0.16, 0.18 |
|  | 80 | 0.27 | 0.26, 0.28 | 0.27 | 0.26, 0.28 | 0.27 | 0.25, 0.28 | 0.27 | 0.25, 0.28 | 0.26 | 0.25, 0.27 | 0.25 | 0.24, 0.26 |
|  | 90 | 0.40 | 0.38, 0.42 | 0.40 | 0.38, 0.42 | 0.39 | 0.37, 0.40 | 0.39 | 0.37, 0.41 | 0.38 | 0.36, 0.39 | 0.36 | 0.35, 0.38 |
|  |  |  |  |  |  |  |  |  |  |  |  |  |  |
| Relative error | 1 | 0.58 | -3.85, 5.01 | 0.57 | -3.86, 4.99 | 0.64 | -3.79, 5.07 | 0.64 | -3.79, 5.07 | 0.76 | -3.68, 5.19 | 0.69 | -3.75, 5.12 |
|  | 5 | 0.74 | -3.70, 5.17 | 0.81 | -3.63, 5.24 | 0.69 | -3.75, 5.12 | 0.62 | -3.81, 5.05 | 0.52 | -3.91, 4.94 | 0.59 | -3.84, 5.02 |
|  | 10 | 1.19 | -3.27, 5.64 | 1.15 | -3.30, 5.61 | 1.01 | -3.44, 5.46 | 0.78 | -3.66, 5.22 | 0.84 | -3.60, 5.29 | 0.82 | -3.62, 5.26 |
|  | 20 | 0.73 | -3.71, 5.16 | 0.98 | -3.47, 5.43 | 0.33 | -4.09, 4.75 | 0.49 | -3.94, 4.91 | 0.18 | -4.23, 4.60 | 0.29 | -4.13, 4.70 |
|  | 40 | 1.55 | -2.93, 6.03 | 1.52 | -2.96, 6.00 | 1.36 | -3.11, 5.83 | 0.46 | -3.98, 4.89 | 1.00 | -3.46, 5.45 | 0.12 | -4.30, 4.54 |
|  | 60 | 1.54 | -2.95, 6.04 | 1.21 | -3.26, 5.69 | 1.76 | -2.74, 6.27 | 0.71 | -3.74, 5.17 | 1.63 | -2.87, 6.13 | 1.24 | -3.25, 5.72 |
|  | 80 | -3.73 | -8.03, 0.58 | -4.01 | -8.31, 0.28 | -4.86 | -9.12, -0.61 | -4.73 | -9.00, -0.47 | -2.29 | -6.66, 2.09 | -3.70 | -8.01, 0.62 |
|  | 90 | -0.77 | -5.35, 3.82 | -2.40 | -6.92, 2.12 | -0.42 | -5.02, 4.18 | -0.87 | -5.44, 3.71 | -2.51 | -7.02, 2.00 | -0.41 | -5.05, 4.22 |
|  |  |  |  |  |  |  |  |  |  |  |  |  |  |
| Coverage | 1 | 94.89 | 93.52, 96.26 | 94.89 | 93.52, 96.26 | 94.99 | 93.64, 96.34 | 94.89 | 93.52, 96.26 | 94.89 | 93.52, 96.26 | 94.99 | 93.64, 96.34 |
|  | 5 | 94.69 | 93.30, 96.08 | 94.79 | 93.41, 96.17 | 94.99 | 93.64, 96.34 | 94.99 | 93.64, 96.34 | 95.19 | 93.86, 96.52 | 94.79 | 93.41, 96.17 |
|  | 10 | 95.49 | 94.20, 96.78 | 95.49 | 94.20, 96.78 | 95.49 | 94.20, 96.78 | 95.19 | 93.86, 96.52 | 95.29 | 93.98, 96.60 | 95.19 | 93.86, 96.52 |
|  | 20 | 95.59 | 94.32, 96.86 | 95.59 | 94.32, 96.86 | 95.29 | 93.98, 96.60 | 95.09 | 93.75, 96.43 | 95.19 | 93.86, 96.52 | 94.69 | 93.30, 96.08 |
|  | 40 | 94.99 | 93.64, 96.34 | 94.99 | 93.64, 96.34 | 95.09 | 93.75, 96.43 | 95.09 | 93.75, 96.43 | 95.39 | 94.09, 96.69 | 94.89 | 93.52, 96.26 |
|  | 60 | 95.49 | 94.20, 96.78 | 95.49 | 94.20, 96.78 | 95.29 | 93.98, 96.60 | 95.59 | 94.32, 96.86 | 94.69 | 93.30, 96.08 | 95.19 | 93.86, 96.52 |
|  | 80 | 95.09 | 93.75, 96.43 | 95.39 | 94.09, 96.69 | 94.89 | 93.52, 96.26 | 94.19 | 92.74, 95.64 | 95.09 | 93.75, 96.43 | 95.19 | 93.86, 96.52 |
|  | 90 | 96.49 | 95.35, 97.63 | 95.99 | 94.78, 97.21 | 95.49 | 94.20, 96.78 | 95.29 | 93.98, 96.60 | 95.09 | 93.75, 96.43 | 95.99 | 94.78, 97.21 |
|  |  |  |  |  |  |  |  |  |  |  |  |  |  |
| % reduction in SE compared to CCA | 1 |  |  | -0.01% |  | 0.10% |  | 0.08% |  | 0.23% |  | 0.19% |  |
|  | 5 |  |  | 0.06% |  | 0.06% |  | 0.01% |  | 0.03% |  | 0.26% |  |
|  | 10 |  |  | -0.01% |  | 0.06% |  | -0.17% |  | 0.15% |  | 0.43% |  |
|  | 20 |  |  | 0.05% |  | 0.00% |  | 0.08% |  | 0.37% |  | 1.07% |  |
|  | 40 |  |  | -0.28% |  | 0.63% |  | 0.09% |  | 0.98% |  | 2.14% |  |
|  | 60 |  |  | -0.18% |  | 1.70% |  | 0.87% |  | 2.51% |  | 4.36% |  |
|  | 80 |  |  | -0.17% |  | 2.45% |  | 2.31% |  | 4.70% |  | 6.90% |  |
|  | 90 |  |  | -0.60% |  | 2.82% |  | 2.27% |  | 5.20% |  | 8.60% |  |

CCA – Complete case analysis; FMI- Fraction of Missing Information; SE – Standard error; R^2^ – the squared coefficient of multiple correlation

^a^ For FMI the estimate was the median across simulations and the interval represents the interquartile range. For all other statistics the mean across simulations was taken and the 95% confidence interval was calculated using Monte Carlo standard error.

Table S13. Performance Statistics for the MAR Results of the Exposure Coefficient in the Binary Outcome Simulation Study.

| Statistic | % missing data | CCA | | Model 1  No aux info, R^2^=0.36 | | Model 2  R^2^=0.40 | | Model 3  R^2^=0.52 | | Model 4  R^2^=0.76 | | Model 5  R^2^=0.92 | |
| --- | --- | --- | --- | --- | --- | --- | --- | --- | --- | --- | --- | --- | --- |
| FMI ^a^ | 1 |  |  | 0.01 | 0.01, 0.02 | 0.01 | 0.01, 0.01 | 0.01 | 0.01, 0.01 | 0.01 | 0.00, 0.01 | 0.01 | 0.00, 0.01 |
|  | 5 |  |  | 0.06 | 0.05, 0.06 | 0.05 | 0.05, 0.06 | 0.03 | 0.03, 0.04 | 0.03 | 0.03, 0.04 | 0.03 | 0.03, 0.04 |
|  | 10 |  |  | 0.11 | 0.10, 0.12 | 0.11 | 0.10, 0.12 | 0.07 | 0.07, 0.08 | 0.07 | 0.06, 0.08 | 0.07 | 0.06, 0.08 |
|  | 20 |  |  | 0.21 | 0.20, 0.23 | 0.21 | 0.19, 0.23 | 0.15 | 0.14, 0.17 | 0.15 | 0.13, 0.16 | 0.14 | 0.12, 0.16 |
|  | 40 |  |  | 0.41 | 0.39, 0.43 | 0.40 | 0.38, 0.42 | 0.32 | 0.30, 0.35 | 0.31 | 0.29, 0.34 | 0.30 | 0.28, 0.33 |
|  | 60 |  |  | 0.60 | 0.58, 0.62 | 0.59 | 0.57, 0.61 | 0.51 | 0.48, 0.54 | 0.51 | 0.47, 0.54 | 0.49 | 0.46, 0.53 |
|  | 80 |  |  | 0.79 | 0.77, 0.81 | 0.79 | 0.76, 0.80 | 0.73 | 0.70, 0.76 | 0.72 | 0.69, 0.75 | 0.71 | 0.68, 0.75 |
|  | 90 |  |  | 0.89 | 0.87, 0.90 | 0.88 | 0.87, 0.90 | 0.85 | 0.82, 0.87 | 0.84 | 0.81, 0.86 | 0.83 | 0.80, 0.86 |
|  |  |  |  |  |  |  |  |  |  |  |  |  |  |
| Bias | 1 | 0.00 | -0.01, 0.00 | 0.00 | -0.01, 0.00 | 0.00 | -0.01, 0.00 | 0.00 | -0.01, 0.01 | 0.00 | -0.01, 0.01 | 0.00 | -0.01, 0.01 |
|  | 5 | -0.01 | -0.02, -0.01 | -0.01 | -0.02, -0.01 | -0.01 | -0.02, -0.01 | 0.00 | -0.01, 0.01 | 0.00 | -0.01, 0.01 | 0.00 | -0.01, 0.01 |
|  | 10 | -0.03 | -0.04, -0.02 | -0.03 | -0.03, -0.02 | -0.03 | -0.03, -0.02 | 0.00 | -0.01, 0.00 | 0.00 | -0.01, 0.00 | 0.00 | -0.01, 0.00 |
|  | 20 | -0.05 | -0.05, -0.04 | -0.05 | -0.05, -0.04 | -0.05 | -0.05, -0.04 | 0.00 | -0.01, 0.00 | 0.00 | -0.01, 0.01 | 0.00 | -0.01, 0.01 |
|  | 40 | -0.08 | -0.09, -0.07 | -0.08 | -0.09, -0.07 | -0.08 | -0.09, -0.07 | -0.01 | -0.02, 0.00 | -0.01 | -0.02, 0.00 | 0.00 | -0.01, 0.00 |
|  | 60 | -0.10 | -0.11, -0.09 | -0.10 | -0.11, -0.09 | -0.10 | -0.11, -0.09 | -0.01 | -0.02, 0.00 | -0.01 | -0.02, 0.00 | -0.01 | -0.02, 0.00 |
|  | 80 | -0.11 | -0.12, -0.10 | -0.11 | -0.13, -0.10 | -0.11 | -0.13, -0.10 | -0.02 | -0.03, 0.00 | -0.01 | -0.03, 0.00 | -0.01 | -0.02, 0.00 |
|  | 90 | -0.09 | -0.11, -0.07 | -0.10 | -0.11, -0.08 | -0.09 | -0.11, -0.08 | -0.01 | -0.03, 0.01 | 0.00 | -0.02, 0.02 | 0.00 | -0.02, 0.01 |
|  |  |  |  |  |  |  |  |  |  |  |  |  |  |
| Empirical SE | 1 | 0.11 | 0.11, 0.12 | 0.11 | 0.11, 0.12 | 0.11 | 0.11, 0.12 | 0.11 | 0.11, 0.12 | 0.11 | 0.11, 0.12 | 0.11 | 0.11, 0.12 |
|  | 5 | 0.11 | 0.11, 0.12 | 0.11 | 0.11, 0.12 | 0.11 | 0.11, 0.12 | 0.11 | 0.11, 0.12 | 0.11 | 0.11, 0.12 | 0.11 | 0.11, 0.12 |
|  | 10 | 0.12 | 0.11, 0.12 | 0.12 | 0.11, 0.12 | 0.12 | 0.11, 0.12 | 0.12 | 0.11, 0.12 | 0.12 | 0.11, 0.12 | 0.12 | 0.11, 0.12 |
|  | 20 | 0.12 | 0.11, 0.13 | 0.12 | 0.12, 0.13 | 0.12 | 0.11, 0.13 | 0.12 | 0.11, 0.12 | 0.12 | 0.11, 0.12 | 0.12 | 0.11, 0.12 |
|  | 40 | 0.13 | 0.13, 0.14 | 0.13 | 0.13, 0.14 | 0.13 | 0.13, 0.14 | 0.13 | 0.13, 0.14 | 0.13 | 0.13, 0.14 | 0.13 | 0.13, 0.14 |
|  | 60 | 0.16 | 0.15, 0.17 | 0.16 | 0.15, 0.17 | 0.16 | 0.15, 0.17 | 0.16 | 0.16, 0.17 | 0.16 | 0.15, 0.17 | 0.16 | 0.15, 0.17 |
|  | 80 | 0.22 | 0.21, 0.23 | 0.22 | 0.21, 0.23 | 0.22 | 0.21, 0.23 | 0.22 | 0.21, 0.23 | 0.22 | 0.21, 0.23 | 0.22 | 0.21, 0.23 |
|  | 90 | 0.30 | 0.29, 0.32 | 0.30 | 0.29, 0.32 | 0.30 | 0.28, 0.31 | 0.31 | 0.29, 0.32 | 0.30 | 0.29, 0.32 | 0.29 | 0.28, 0.30 |
|  |  |  |  |  |  |  |  |  |  |  |  |  |  |
| Relative error | 1 | 0.73 | -3.70, 5.16 | 0.67 | -3.76, 5.10 | 0.71 | -3.72, 5.14 | 0.81 | -3.63, 5.25 | 0.77 | -3.66, 5.21 | 0.76 | -3.67, 5.20 |
|  | 5 | 0.86 | -3.57, 5.30 | 0.84 | -3.60, 5.27 | 0.93 | -3.51, 5.37 | 1.20 | -3.26, 5.65 | 1.19 | -3.27, 5.64 | 1.13 | -3.32, 5.58 |
|  | 10 | 0.49 | -3.94, 4.91 | 0.56 | -3.86, 4.99 | 0.41 | -4.01, 4.83 | 0.77 | -3.67, 5.20 | 0.54 | -3.88, 4.97 | 0.42 | -4.00, 4.85 |
|  | 20 | 2.03 | -2.46, 6.52 | 1.99 | -2.50, 6.48 | 1.89 | -2.59, 6.38 | 2.41 | -2.10, 6.92 | 2.26 | -2.25, 6.76 | 2.11 | -2.39, 6.61 |
|  | 40 | 1.56 | -2.91, 6.04 | 1.91 | -2.58, 6.40 | 2.44 | -2.08, 6.95 | 1.91 | -2.58, 6.40 | 2.32 | -2.18, 6.83 | 1.97 | -2.52, 6.47 |
|  | 60 | 0.19 | -4.23, 4.61 | 0.37 | -4.06, 4.79 | -0.26 | -4.67, 4.14 | -0.43 | -4.82, 3.96 | -1.11 | -5.47, 3.26 | -1.29 | -5.65, 3.07 |
|  | 80 | -1.58 | -5.94, 2.78 | -0.94 | -5.33, 3.46 | -2.26 | -6.60, 2.08 | -1.28 | -5.65, 3.10 | -1.84 | -6.20, 2.51 | -3.00 | -7.31, 1.31 |
|  | 90 | -1.45 | -5.86, 2.97 | -1.18 | -5.61, 3.25 | -1.57 | -5.99, 2.85 | -2.17 | -6.56, 2.21 | -2.45 | -6.83, 1.93 | -0.54 | -5.01, 3.93 |
|  |  |  |  |  |  |  |  |  |  |  |  |  |  |
| Coverage | 1 | 95.09 | 93.75, 96.43 | 95.09 | 93.75, 96.43 | 94.89 | 93.52, 96.26 | 95.09 | 93.75, 96.43 | 94.89 | 93.52, 96.26 | 94.89 | 93.52, 96.26 |
|  | 5 | 94.99 | 93.64, 96.34 | 94.99 | 93.64, 96.34 | 94.99 | 93.64, 96.34 | 95.19 | 93.86, 96.52 | 95.29 | 93.98, 96.60 | 95.39 | 94.09, 96.69 |
|  | 10 | 94.39 | 92.96, 95.82 | 94.49 | 93.07, 95.90 | 94.29 | 92.85, 95.73 | 95.09 | 93.75, 96.43 | 95.29 | 93.98, 96.60 | 95.29 | 93.98, 96.60 |
|  | 20 | 93.89 | 92.40, 95.37 | 93.79 | 92.29, 95.29 | 93.89 | 92.40, 95.37 | 94.99 | 93.64, 96.34 | 95.49 | 94.20, 96.78 | 95.49 | 94.20, 96.78 |
|  | 40 | 91.08 | 89.31, 92.85 | 90.68 | 88.88, 92.48 | 90.48 | 88.66, 92.30 | 95.39 | 94.09, 96.69 | 95.89 | 94.66, 97.12 | 95.69 | 94.43, 96.95 |
|  | 60 | 88.88 | 86.93, 90.83 | 88.58 | 86.60, 90.55 | 87.88 | 85.85, 89.90 | 94.79 | 93.41, 96.17 | 94.79 | 93.41, 96.17 | 93.89 | 92.40, 95.37 |
|  | 80 | 90.28 | 88.44, 92.12 | 89.88 | 88.01, 91.75 | 90.28 | 88.44, 92.12 | 94.39 | 92.96, 95.82 | 94.59 | 93.19, 95.99 | 94.09 | 92.62, 95.55 |
|  | 90 | 93.59 | 92.07, 95.11 | 93.09 | 91.51, 94.66 | 92.99 | 91.40, 94.57 | 95.19 | 93.86, 96.52 | 94.49 | 93.07, 95.90 | 94.59 | 93.19, 95.99 |
|  |  |  |  |  |  |  |  |  |  |  |  |  |  |
| % reduction in SE compared to CCA | 1 |  |  | -0.05% |  | 0.00% |  | 0.18% |  | 0.15% |  | 0.16% |  |
|  | 5 |  |  | -0.02% |  | 0.17% |  | 0.69% |  | 0.72% |  | 0.72% |  |
|  | 10 |  |  | 0.01% |  | -0.07% |  | 0.78% |  | 0.72% |  | 0.72% |  |
|  | 20 |  |  | -0.15% |  | 0.01% |  | 0.92% |  | 1.03% |  | 1.22% |  |
|  | 40 |  |  | 0.09% |  | 1.27% |  | 0.63% |  | 1.53% |  | 1.72% |  |
|  | 60 |  |  | -0.10% |  | 0.22% |  | -1.25% |  | -1.09% |  | -0.10% |  |
|  | 80 |  |  | 0.39% |  | 0.48% |  | -0.73% |  | -0.20% |  | 0.26% |  |
|  | 90 |  |  | -0.07% |  | 1.58% |  | -1.63% |  | 0.01% |  | 4.15% |  |
|  |  |  |  |  |  |  |  |  |  |  |  |  |  |
| % reduction in bias compared to CCA | 1 |  |  | 2.51% |  | 3.72% |  | 86.84% |  | 90.22% |  | 93.93% |  |
|  | 5 |  |  | -2.52% |  | -6.71% |  | 88.27% |  | 91.64% |  | 93.16% |  |
|  | 10 |  |  | 0.77% |  | 2.52% |  | 86.72% |  | 85.34% |  | 88.59% |  |
|  | 20 |  |  | -0.19% |  | 0.42% |  | 91.25% |  | 95.04% |  | 97.80% |  |
|  | 40 |  |  | -4.20% |  | -2.67% |  | 89.05% |  | 91.25% |  | 93.78% |  |
|  | 60 |  |  | -2.47% |  | -3.38% |  | 88.28% |  | 90.88% |  | 93.12% |  |
|  | 80 |  |  | -3.77% |  | -3.12% |  | 83.97% |  | 86.65% |  | 89.63% |  |
|  | 90 |  |  | -3.11% |  | -1.05% |  | 91.36% |  | 96.95% |  | 95.96% |  |

CCA – Complete case analysis; FMI- Fraction of Missing Information; SE – Standard error; R^2^ – the squared coefficient of multiple correlation

^a^ For FMI the estimate was the median across simulations and the interval represents the interquartile range. For all other statistics the mean across simulations was taken and the 95% confidence interval was calculated using Monte Carlo standard error.


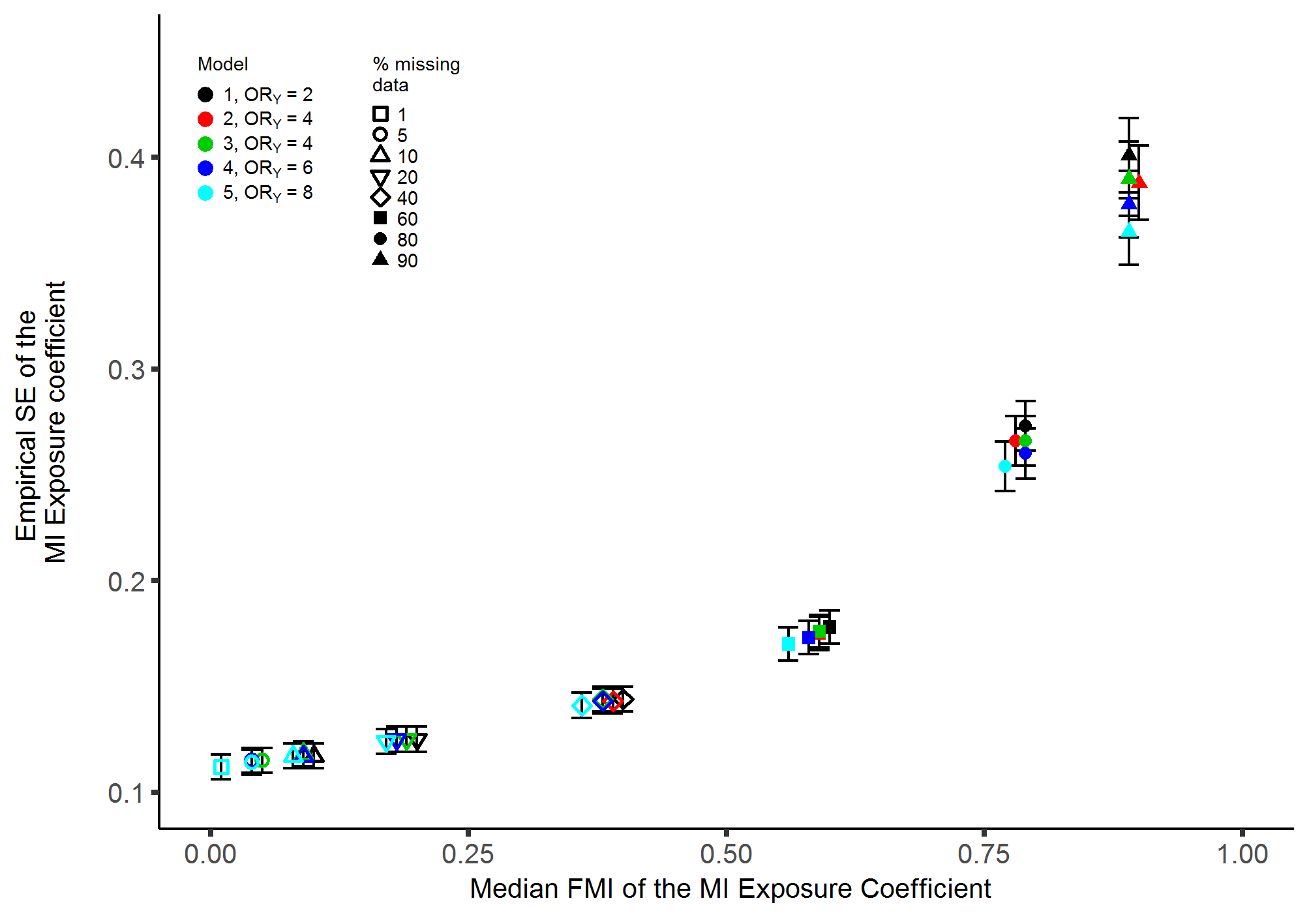


Figure S4: Empirical SE of the MI exposure coefficient plotted against FMI for simulated MCAR binary outcome data. Error bars are 95% confidence intervals based on Monte Carlo standard errors across simulations. FMI = fraction of missing information; SE = standard error.


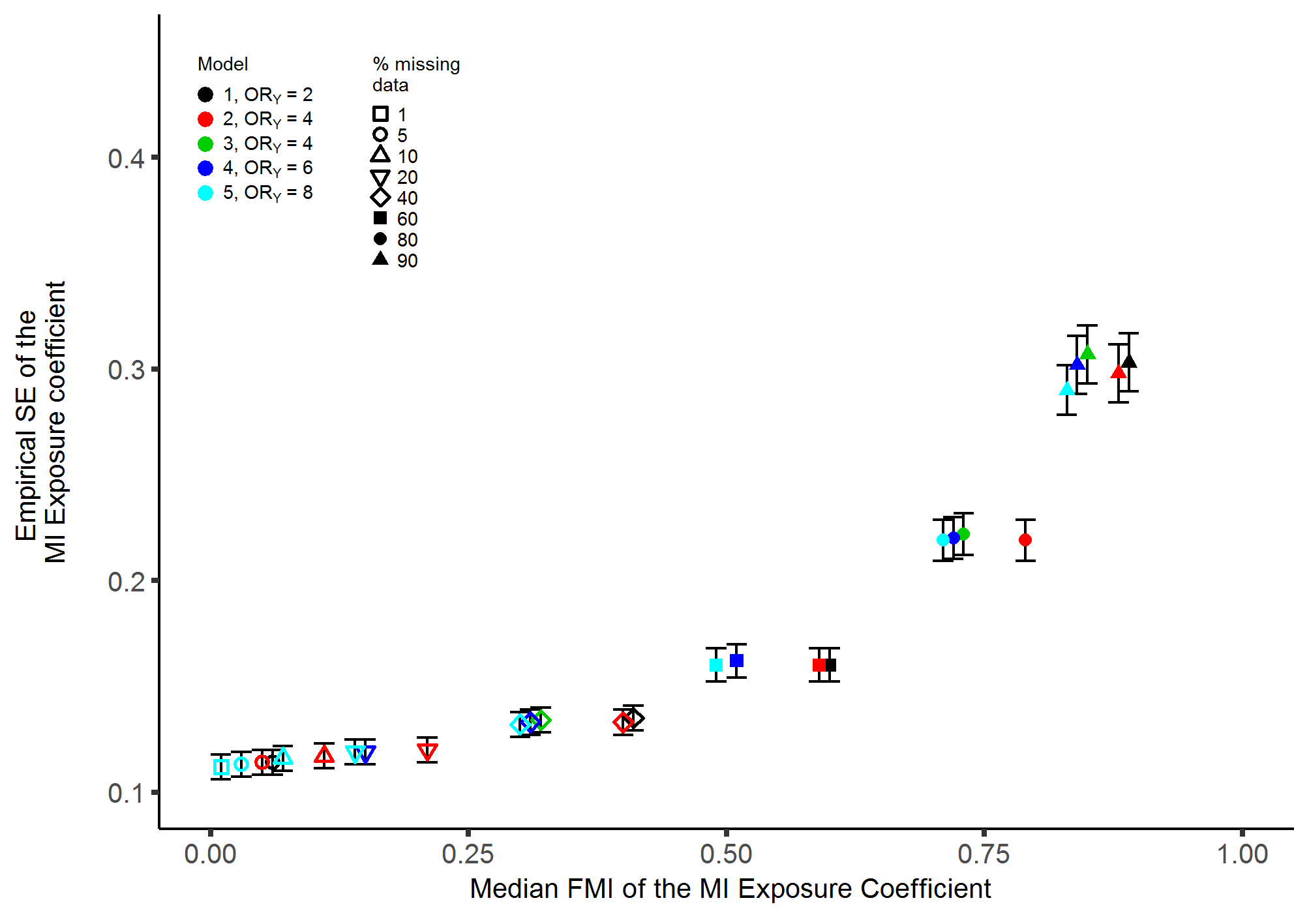


Figure S5: Empirical SE of the MI exposure coefficient plotted against FMI for simulated MAR binary outcome data. Error bars are 95% confidence intervals based on Monte Carlo standard errors across simulations. FMI = fraction of missing information; SE = standard error.
